# Selective Serotonin Reuptake Inhibitor (SSRI) Antidepressants in Pregnancy and Congenital Anomalies: Analysis of Linked Databases in Wales, Norway and Funen, Denmark

**DOI:** 10.1371/journal.pone.0165122

**Published:** 2016-12-01

**Authors:** Sue Jordan, Joan K. Morris, Gareth I. Davies, David Tucker, Daniel S. Thayer, Johannes M. Luteijn, Margery Morgan, Ester Garne, Anne V. Hansen, Kari Klungsøyr, Anders Engeland, Breidge Boyle, Helen Dolk

**Affiliations:** 1 Department of Nursing, College of Human and Health Sciences, Swansea University, Swansea, United Kingdom; 2 Queen Mary University of London, London, United Kingdom; 3 Public Health Wales, Swansea, United Kingdom; 4 Paediatric Department, Hospital Lillebaelt, Kolding, Denmark; 5 Department of Public Health, University of Copenhagen, Copenhagen, Denmark; 6 Department of Global Public Health and Primary Care, University of Bergen, Bergen, Norway; 7 Department of Health Registries, Norwegian Institute of Public Health, Bergen, Norway; 8 Department of Pharmacoepidemiology, Norwegian Institute of Public Health Bergen, Bergen, Norway; 9 Centre for Maternal, Fetal and Infant Research, Ulster University, Newtownabbey, Co Antrim, Northern Ireland, United Kingdom; Chiba Daigaku, JAPAN

## Abstract

**Background:**

Hypothesised associations between in utero exposure to selective serotonin reuptake inhibitors (SSRIs) and congenital anomalies, particularly congenital heart defects (CHD), remain controversial. We investigated the putative teratogenicity of SSRI prescription in the 91 days either side of first day of last menstrual period (LMP).

**Methods and Findings:**

Three population-based EUROCAT congenital anomaly registries- Norway (2004–2010), Wales (2000–2010) and Funen, Denmark (2000–2010)—were linked to the electronic healthcare databases holding prospectively collected prescription information for all pregnancies in the timeframes available. We included 519,117 deliveries, including foetuses terminated for congenital anomalies, with data covering pregnancy and the preceding quarter, including 462,641 with data covering pregnancy and one year either side. For SSRI exposures 91 days either side of LMP, separately and together, odds ratios with 95% confidence intervals (ORs, 95%CI) for all major anomalies were estimated. We also explored: pausing or discontinuing SSRIs preconception, confounding, high dose regimens, and, in Wales, diagnosis of depression. Results were combined in meta-analyses. SSRI prescription 91 days either side of LMP was associated with increased prevalence of severe congenital heart defects (CHD) (as defined by EUROCAT guide 1.3, 2005) (34/12,962 [0.26%] vs. 865/506,155 [0.17%] OR 1.50, 1.06–2.11), and the composite adverse outcome of 'anomaly or stillbirth' (473/12962, 3.65% vs. 15829/506,155, 3.13%, OR 1.13, 1.03–1.24). The increased prevalence of all major anomalies combined did not reach statistical significance (3.09% [400/12,962] vs. 2.67% [13,536/506,155] OR 1.09, 0.99–1.21). Adjusting for socio-economic status left ORs largely unchanged. The prevalence of anomalies and severe CHD was reduced when SSRI prescriptions were stopped or paused preconception, and increased when >1 prescription was recorded, but differences were not statistically significant. The dose-response relationship between severe CHD and SSRI dose (meta-regression OR 1.49, 1.12–1.97) was consistent with SSRI-exposure related risk. Analyses in Wales suggested no associations between anomalies and diagnosed depression.

**Conclusion:**

The additional absolute risk of teratogenesis associated with SSRIs, if causal, is small. However, the high prevalence of SSRI use augments its public health importance, justifying modifications to preconception care.

## Introduction

Exposure to selective serotonin reuptake inhibitors (SSRIs) during the first trimester of pregnancy, including the crucial period of organogenesis (the first 49 days after implantation)[1], affects 4% of pregnant women in the USA[2] and UK[3]. SSRI prescribing indications, mainly depression, panic, obsessive-compulsive or social anxiety disorders, and, for fluoxetine, bulimia nervosa, are not always recorded [3]. SSRIs, particularly fluoxetine and citalopram, and their metabolites, cross the placenta[4], and appear in cord blood[5,6]; their presence in amniotic fluid prolongs foetal exposure. SSRIs, and some other antidepressants, act on the crucial serotonin transporter (SERT, aka 5HTT, SLC6A4, OMIM 182138), which regulates the synaptic concentration of serotonin (5HT) in many tissues, including the placenta[7]. The resultant increased bioavailability of serotonin affects vasoconstriction and coagulation or bruising [6,8,9], cardiac morphogenesis [10,11], CNS development[6] gastrulation, laterality and craniofacial development[10], conferring biological plausibility on reported associations between SSRI exposure during organogenesis and certain congenital anomalies.

The full impact of exposure to SSRIs *in utero* is incompletely understood, and not all problems initially suspected [12] have been confirmed by further investigation. Some[13–16], but not all[17–22], observational studies indicate significant associations between SSRI exposure during organogenesis and all congenital anomalies combined. Risks may be confined to specific SSRIs and specific anomalies[23,24]. However, the literature offers no consistency: paroxetine is implicated in some studies[24],[25], and fluoxetine[24,26,27], citalopram/ escitalopram[17,27] and sertraline[17,28] in others. Meta-analyses[26,29,30] and analysis of 12 EUROCAT registries[31] indicate an overall association between SSRI exposure and congenital heart defects (CHD); however, there is no consensus[21,22,29,32,33]. The most persistent associations relate to paroxetine exposure and CHD[22,24,27,30,31], particularly at doses >25mg/day[34]. Epidemiologists also report increased risks of: neural tube defects[33,35], ano-rectal stenosis/ atresia[23], gastroschisis, omphalocele[35], renal dysplasia, hypospadias[27], limb reduction[23], talipes equinovarus (clubfoot)[23], craniosynostosis[35], anomalies of the eye[18], ear, face[36], respiratory[36] and digestive tracts[15,24].

To investigate the putative teratogenicity of SSRIs, three countries from the pan-European congenital anomalies registry network[37,38] were linked with healthcare databases. We aimed to examine any associations between major congenital anomalies and: prescription of antidepressant medicines in the 91 days either side of the 1^st^ day of last menstrual period (LMP); high dose SSRI regimens; confounding; pausing or stopping SSRI pharmacotherapy before pregnancy; and diagnosed, unmedicated depression.

## Methods

Three population-based cohorts containing prospectively collected linked prescription data were interrogated using a common protocol. Ethical and data access approvals were obtained for each country from the relevant governance infrastructures (see acknowledgements).

### Settings

Three congenital anomalies registries that contribute to EUROCAT[37,39] were linked with prescription and healthcare databases covering their source populations[40,41]. We examined anonymised linked routinely collected data on congenital anomalies, primary care prescribing (Wales) or dispensing (Denmark, Norway), concurrent maternal diagnoses and demographic indicators from:

1. Denmark’s Medical Birth registry, Danish national Prescription and Patient registers, Statistics Denmark[42] and the Funen, Denmark (Odense) EUROCAT register.
2. Norway’s Medical Birth Registry, containing all EUROCAT cases, linked to the National Prescription Database and the National Education Database[43,44].
3. Wales’ health and social care linked electronic databank (the Secure Anonymised Information Linkage [SAIL]). SAIL links primary care records, including prescriptions, for ~40% of the population to the Office of National Statistics births and deaths register, the National Community Child Health Database (NCCHD), the Patient Episode Database for Wales and CARIS (Congenital Anomaly Register and Information Service for Wales). All general practices were invited to participate, without payment, and ~40% had done so in 2014[45,46].

Databases were linked by trusted third parties (Statistics Denmark, Statistics Norway in conjunction with the National Prescription Database, NHS Wales Informatics Service) using unique personal identifiers, which remained undisclosed to researchers, ensuring anonymity.

The 3 countries have similar population sizes and life expectancies, but differ in: GDP *per capita* (Wales $23.90k [47] Denmark $50.46, Norway $72.96K[48]; the proportion of children living in poverty, defined as <60% median household income (Wales 28% [49], Denmark 7% and Norway 8% [50]); and unemployment rates (Wales 5.3%, Denmark 3.9% and Norway 3.4%)[47,48], (2006 data). Much of Wales is an EU Convergence area [51].

### Study population

The study population included all foetuses and infants who 1) would have appeared in the EUROCAT registries had they been diagnosed with a major congenital anomaly, 2) had linked maternal prescription data, and 3) whose birth outcome was either live birth or still birth/ late foetal death after 20 weeks (24 weeks in Wales) or termination of pregnancy for foetal anomaly (TOPFA) recorded in the EUROCAT register. Deliveries from 1^st^ January 2000 to 31^st^ December 2010 were included in Wales and Denmark. In Norway, all pregnancies with date of LMP after April 1^st^ 2004 and ending before 31^st^ December 2010 were included, to coincide with the start of the prescription database. In Wales, infants were included where the associated maternal ID was in the geographical areas that could be linked with the primary care dataset and the record was complete[41]. We prepared 2 datasets with an inclusion criterion relating to the woman’s time on the linked database with prescription information:

1. 91 days before LMP to delivery (birth or TOPFA) (main analysis)
2. 365 days before LMP to 365 days after delivery

Information on start of pregnancy was obtained from ultrasound scan data recorded in the Medical Birth Registers of Norway and Denmark (MBRN, MBRD), and the NCCHD for Wales [41]. Representivity was checked in Denmark and Wales by comparing socioeconomic status (SES) with national populations.

### Exposure

Exposure was defined as one or more prescription for an antidepressant issued (Wales) or dispensed (Norway & Denmark) 91 days either side of the first day of LMP. We based our timeframe on prescription duration (typically 90 days) and relevant pharmacokinetic parameters: for example elimination of the active metabolite of fluoxetine can take ~40 days in adults[52], and longer in the embryo or foetus[11]. Antidepressants were investigated according to anatomical, therapeutic, chemical (ATC) classification [53]: 1) grouped a) all SSRIs (NO6AB); b) all antidepressants (NO6A); 2) as individual SSRIs, fluoxetine, citalopram, paroxetine, sertraline, escitalopram, fluvoxamine. Where more than one SSRI had been prescribed, exposure was not allocated to either SSRI; women switching were retained as SSRI exposed. Where non-SSRI antidepressants were co-prescribed, exposure was classified according to the SSRI. Denmark supplied data on SSRIs only.

**Dose** was calculated from tablet and capsule sizes, to avoid missing data. We classified high dose exposure as prescription of: 60mg fluoxetine, 40mg citalopram, 30mg paroxetine, 100mg sertraline, 20mg escitalopram, based on tablet/ capsule sizes quoted in the British National Formulary (BNF)[54]. Smaller tablets and capsules were classified as ‘other dose’ (low or medium).

### Outcomes

Major congenital anomalies were classified according to the EUROCAT standard subgroups, as defined in EUROCAT Guide 1.3, chapter 2.2 [37]. Severe CHD was defined as ICD10 codes: Q200 (common arterial trunk), Q203 (discordant ventriculoarterial connection), Q204 (double inlet ventricle), Q212 (atrio-ventricular septal defect), Q2121 (primum atrial septal defect [ASD]), Q213 (tetralogy of Fallot), Q220 (pulmonary valve atresia), Q224 (tricuspid stenosis or atresia), Q225 (Ebstein’s anomaly), Q226 (hypoplastic right heart), Q230 (stenosis or atresia of aortic valve), Q234 (hypoplastic left heart), Q251 (co-arctation of aorta), Q262 (total anomalous pulmonary venous connection). Patent ductus arteriosus in pre-term infants was not included as CHD. Minor anomalies are not recorded in EUROCAT, and not investigated. Congenital anomaly cases, diagnosed within the first year of life, irrespective of mother’s time on database, were as reported to EUROCAT: October 2014 (Wales), February 2012 (Denmark), February 2014 (Norway). We excluded, from the main analysis, subjects with anomalies of chromosomal (EUROCAT subgroup al88) or genetic (al104, al105 & al108) aetiology, including sequences[37].

We analysed as prior hypotheses associations between SSRI prescription and 10 pre-specified anomalies identified from the literature as associated with SSRI exposure[31]: CHD, severe CHD, neural tube defects, ano-rectal atresia/stenosis, renal dysplasia, craniosynostosis, hypospadias, including 3 anomalies associated with vasoconstriction (limb reduction, abdominal wall defects (gastroschisis and omphalocele) [55,56], and talipes equinovarus[57]. ^e^ Abdominal wall defects (gastroschisis, Q792, omphalocele, Q793 and other wall defects, Q795) were combined to achieve sufficient numbers to report, considering their purported common aetiology (vasoconstriction of the omphalomesenteric artery) [58,59].

The composite outcomes, all major anomalies combined and major congenital anomalies or stillbirth, were based on the ICH (International Conference on Harmonisation) definition of serious adverse events[60].

### Confounding

To minimise **confounding by co-exposure**, we achieved a relatively homogeneous population by excluding infants: 1) with EUROCAT coding[37] indicating known teratogenic syndromes (EUROCAT subgroups al82-84, al86) 2) exposed to medicines more closely associated with congenital anomalies than SSRIs during the 91 days either side of 1^st^ day of LMP: anti-epileptic drugs (AEDs) (NO3)[61]; coumarins (B01AA), mainly warfarin[62]; insulins (A10A)[63]. We examined, but did not exclude, SSRI exposed cases for: 1) exposure to other potentially teratogenic prescription medicines 91 days either side of 1^st^ day of LMP: systemic isotretinoin (D10BA); angiotensin converting enzyme inhibitors or angiotensin II blockers (C09); lithium (NO5AN); benzodiazepines (N05BA); first generation antipsychotics (N05AA through N05AG); second generation antipsychotics (N05AH, N05AL, N05AX); carbimazole (H03BB); thyroxine (N03AA); medicines rarely prescribed in primary care but associated with anomalies: aminoglycosides, ergot derivatives, lindane, gold salts, penicillamine, methotrexate, chloroquine, radiopharmaceuticals[64]; 2) heavy alcohol use and substance misuse (Wales only); 3) maternal conditions indicating that the woman might not be considered to be from the normal healthy population: hospital admission for cancer; thyroid disorders; phenylketonuria; maternal congenital anomalies[65]; 4) maternal siblings with anomalies.

To explore **confounding by indication** (usually depressive illness [66]) we investigated whether women who discontinued prescriptions before the time when a biological effect would be expected (91 days before LMP) had similar risks to those receiving SSRI prescriptions during the vulnerable period (91 days either side of LMP). Those who discontinued were divided into those who did and did not resume prescriptions within a year of delivery. We defined:

- ‘paused SSRI exposure’ as ≥1 prescription during the 3–12 months before pregnancy plus ≥1 prescription during the year after pregnancy and no prescriptions during both the quarter preceding pregnancy and pregnancy.
- discontinuation (stopping) as ≥1 prescription during the 3–12 months before pregnancy and no further prescriptions throughout the quarter preceding pregnancy, pregnancy, and the first year after delivery.

As a sensitivity analysis, we repeated this analysis defining exposure as >1 prescription in each time period (1 year to 91 days before LMP, LMP ± 91 days, 1 year after delivery).

### Statistical analysis

For each country separately, we explored associations between pre-specified outcomes (above) plus each congenital anomaly subgroup and all SSRIs, individual SSRIs, and all antidepressants. For exploration of individual SSRIs, those exposed to other SSRIs were excluded from the analysis. The odds of exposure for subjects with and without each anomaly were compared by calculating odds ratios (ORs) with Cornfield 95% confidence intervals (95% CI). For anomalies with >2 exposed cases in the 3 countries combined, meta-analysis of country-specific effects was undertaken, using the Mantel Haenszel method, with alternative continuity corrections, described by Sweeting *et al*. (2004)[67]. Heterogeneity was assessed using the *I*^*2*^ statistic. We repeated the analysis of all SSRIs excluding infants exposed to any non-SSRI antidepressants (e.g. SNRIs, tricyclic antidepressants); data availability restricted this to Norway & Wales. When evaluating associations other than the 10 pre-specified signals, we applied Simes’ multiple testing procedure to control the false discovery rate to 5% (FDR) [68,69]. For ‘all anomalies’, ‘anomalies + stillbirths’, CHD, and severe CHD, confounding by smoking and socio-economic status were explored in separate fixed effects logistic regression models. The SSRI dose-response relationships for all anomalies, CHD, severe CHD and ‘anomaly or stillbirth’ were explored for zero, ‘other’ and high doses, using random effects meta-regression. Analyses were performed using Stata 12.1[70].

### Wales sub-cohort

In Wales, confounding by indication was further explored by investigating depression and unmedicated depression, defined as a diagnosis of depression in the woman’s record any time during her registration with a participating GP before the end of the first trimester, but no antidepressant prescribed in the 91 days either side of the 1^st^ day of LMP. We explored associations between prespecified anomalies [31], and socioeconomic status (as Townsend fifth), smoking, antipsychotics, substance misuse and heavy drinking and Down syndrom [71] in *a posteriori* subgroups, to generate hypotheses for future work. Substance misuse often coincides with heavy drinking, and *vice versa*, and we combined the two exposures. We took recorded diagnoses of misuse at any time as indicative of a problem likely to recur. Analyses were undertaken in SPSS version 20 for Windows[72].

## Results

**Fig 1:**
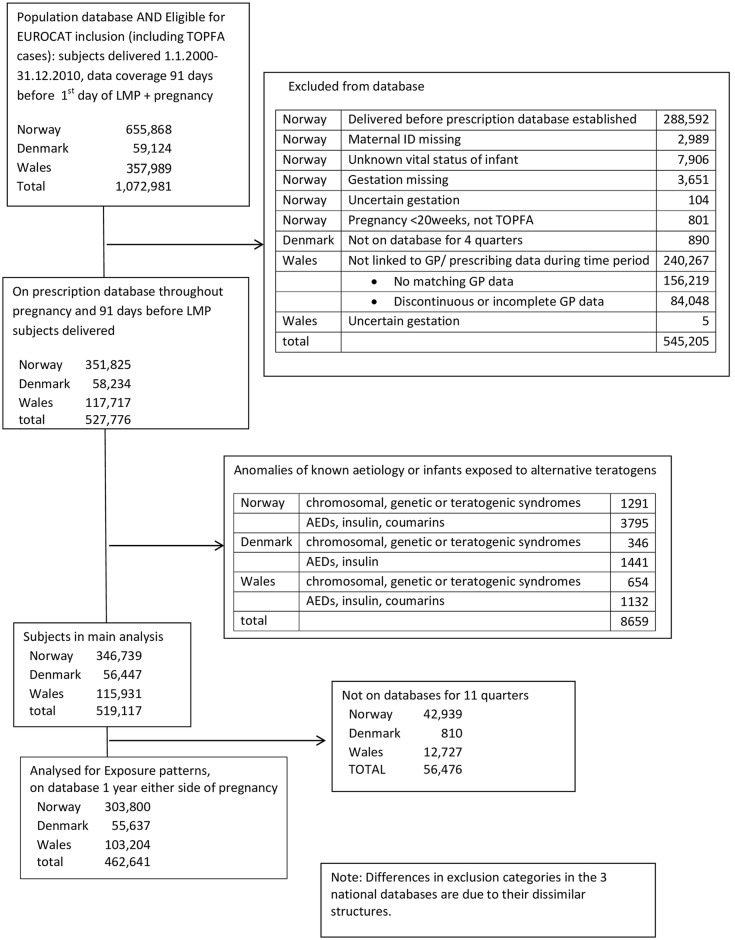
Participant Flow diagram.

The population comprised 519,117 subjects (foetuses and infants): 346,739 from Norway; 56447 from Funen, Denmark; 115,931 from Wales (Tables Aa-Ac in S1 Appendix, Fig 1). In Wales, the included population were less deprived than the rest of Wales [41]. There were no significant demographic differences between Funen County and ‘all Denmark’. Exposure to SSRIs and antidepressants and prevalence of non-chromosomal, non-genetic congenital anomalies were higher in Wales than the Scandinavian countries (Table 1). Norway had the lowest prescription rates for paroxetine, >1 type of SSRI and high doses (Table 2).

**Table 1:** Summary of SSRI and antidepressant exposures and congential anomaly (CA) prevalence in 3 countries: Denmark, Norway, Wales.

	Total Number			SSRI exposed^a^ LMP+/-91 days^b^				Antidepressant exposed LMP+/-91 days			
	Pop (N)	CA Cases (N)	Prevalence of CA (%)	Pop (N)	CA Cases (N)	% of population exposed	Prevalence of CA (%)	Pop (N)	CA Cases (N)	% of population exposed	Prevalence of CA (%)
Denmark	56,447	1288	2.28	1169	33	2.07	2.82	no data	no data		
Norway	346,739	8991	2.59	5451	149	1.57	2.73	7619	198	2.20	2.60
Wales	115,931	3657	3.15	6342	218	5.47	3.44	8019	264	6.92	3.29
Total	519,117	13,936	2.68	12,962	400	2.50	3.09	15,638	462	3.01	2.95

^a^Exposure defined as >0 prescriptions of SSRIs at any dose with or without co-prescriptions.

^b^LMP+/-91 days represents 91 days either side of 1^st^ day of LMP.

Exclusions: 1) chromosomal, genetic, teratogenic anomalies; 2) exposure during the 91 days either side of LMP to: insulin, AEDs, coumarins.

**Table 2:** SSRI exposure 91 days either side of LMP^a^ by individual SSRI^b^ and dose: 3 countries.

		Denmark		Norway		Wales		Summed	
		Number exposed	% total exposed	Number exposed	% total exposed	Number exposed	% total exposed	Number exposed	% total exposed
Population		56,447		346,739		115,931		519,117	
Exposed to any SSRI		1169	100	5451	100	6342	100	12,962	100
SSRI	Fluoxetine	155	13.26	509	9.34	1937	30.54	2601	20.07
	Citalopram	478	40.89	867	15.91	2683	42.31	4028	31.08
	Paroxetine	106	9.07	325	5.96	638	10.06	1069	8.25
	Sertraline	175	14.97	804	14.75	395	6.23	1374	10.60
	Escitalopram	138	11.80	2750	50.45	348	5.49	3236	24.97
	Exposed >1 SSRI	117	10.01	196	3.60	341	5.38	654	5.05
Dose	High	255	21.81	364	6.68	810	12.77	1429	11.02
	Other	914	78.19	5087	93.32	5532	87.23	11,533	88.98

^a^Exclusions and exposures as Table 1.

^b^Fluvoxamine: 28 exposures and 0 exposed cases were identified, see Table Ba in S1 Appendix.

The prevalence of **major congenital anomalies** was higher amongst those exposed to **SSRI** prescriptions 91 days either side of 1^st^ day of LMP (3.09%) than those unexposed (2.67%); however, this was not statistically significant (OR 1.09, 0.99–1.21, Table 3). Exposure was significantly associated with the composite adverse outcome ‘any major anomaly or stillbirth’ (OR 1.13, 1.03–1.24, [Table 3], number needed to harm [NNH] 192, 95% CI 118–512), severe CHD (OR 1.50, 1.06–2.11, NNH 1094, 555–38,141), and abdominal wall defects (OR 1.75, 1.07–2.88, NNH 1629, 832–39,830).

**Table 3:** SSRI (NO6AB) exposures 91 days either side of LMP^a^ and outcomes^b^based on signals: 3 countries.

	Denmark				Norway				Wales				Summed					
	exposed n = 1169		unexposed n = 55,278		exposedn = 5451		unexposed n = 341,288		exposedn = 6342		Unexposed n = 109,589		exposed n = 12962		unexposed n = 506,155		Meta-analysis	
	n	%	N	%	n	%	N	%	n	%	n	%	n	%	n	%	OR, 95% CIs	I^2^
Anomaly or stillbirth	40	3.42	1586	2.87	185	3.39	10412	3.05	248	3.91	3819	3.48	473	3.65	15829	3.13	**1.13 (1.03–1.24)**	0
All Anomalies	33	2.82	1255	2.27	149	2.73	8842	2.59	218	3.44	3439	3.14	400	3.09	13,536	2.67	1.09 (0.99–1.21)	0
Neural Tube Defects	<5		55		6	0.11	271	0.08	10	0.16	123	0.11	17–20	0.14	449	0.09	1.43 (0.89–2.30)	0
CHD	16	1.37	447	0.81	44	0.81	3027	0.89	61	0.96	1029	0.94	121	0.93	4503	0.89	1.03 (.86–1.24)	55.66
Severe CHD	6	0.51	98	0.18	9	0.17	567	0.17	19	0.30	200	0.18	34	0.26	865	0.17	**1.50 (1.06–2.11)**	51.36
Abdominal wall defects^c^^,^	<5		18–21		6	0.11	174	0.05	8	0.13	80	0.07	15–18	0.12	275	0.05	**1.92 (1.13–3.24)**	0
Talipes equinovarus ^c^	<5		73–76		12	0.22	473	0.14	11	0.17	190	0.17	24–27	0.19	736–9	0.15	1.20 (0.79–1.8)	0
Hypospadias	<5		119–122		12	0.22	726	0.21	21	0.33	295	0.27	34–37	0.28	1140–1143	0.23	1.15 (0.82–1.61)	0
Ano-rectal atresia and stenosis^d^													7	0.06	150	0.03	1.85 (0.86–3.96)	0
Renal Dysplasia ^d^													10	0.08	177	0.03	1.57 (0.83–2.98)	0
Limb reduction ^c^^,^ ^d^													6	0.05	254	0.05	0.81 (0.36–1.82)	0
Craniosynostosis ^d^													4	0.03	115	0.02	0.81 (0.3–2.21)	0

We are unable to disclose numbers 1–4 from any single country. Accordingly, we are only able to supply ranges for related values. Where countries combined had <5 exposed cases we report only as an aggregate.

^a^Exclusions and exposures as Table 1.

^b^Anomalies selected for reporting based on background literature[31].

^c^Anomalies associated with vasoconstriction [55].

^d^Data from each country were analysed separately, but low numbers preclude reporting by country for these anomalies plus gastroschisis and omphalocele.

Further information is in Table Ba, Bb (including numbers and %s of cases), and Table C in S1 Appendix and EMC 2015 supplementary tables S3 and S4 [41].

Analyses of SNRI exposure in Wales and Norway are in Table Bb and EMC (2015) [41] (Denmark was unable to supply data on SNRIs). There were 1448 SNRI exposures and 46 exposed cases (3.18%) (OR 1.14, 0.85–1.53). No associations with anomalies listed above where 95% confidence intervals did not include one were identified.

Emboldened text indicates 95% confidence intervals exclude 1.

CHD represents congenital heart defect.

We did not confirm associations between SSRIs and all CHD, neural tube defects, talipes equinovarus, hypospadias, renal dysplasia, ano-rectal atresia/stenosis, limb reduction or craniosynostosis. The association with gastroschisis did not reach statistical significance (OR 1.92, 0.97–3.78, based on 9 exposed cases, Table C in S1 Appendix). Non-significant positive associations involved all individual SSRIs, and included paroxetine with all CHD and ventricular septal defect (VSD), fluoxetine with neural tube defects and citalopram with hypospadias. Escitalopram was associated with talipes equinovarus and abdominal wall defects (Tables Ba, C in S1 Appendix). For **all antidepressants**, differences in prevalence of major anomalies between exposed and unexposed were less marked and not statistically significant (OR 1.03, 0.93–1.13) (Tables Bb, C in S1 Appendix). There were <3 exposed cases for 27/75 anomalies (Tables Ba, Bb in S1 Appendix). When Simes’ False Discovery Rate procedure was applied, no associations reached the 5% false discovery rate significance level threshold, including those with diaphragmatic hernia and syndactyly (Table D in S1 Appendix). When data were re-analysed in Norway and Wales, with those **co-prescribed other antidepressants excluded**, prevalence rates and ORs changed little (Table C in S1 Appendix). Heterogeneity was low for most outcomes, except CHD and severe CHD (Table 3). The NNH for severe CHD varied: 298 (172–11,111) in Denmark, 854 (400–14,286) in Wales, and there was no association in Norway.

### Dose-response

A minority were exposed to prescriptions for high doses of SSRIs (Table 2). Meta-regression for the 3 categories (high, other and zero dose) indicated significant associations for severe CHD and ‘anomaly or stillbirth’ and non-significant trends for all anomalies and CHD (Table 4). Denmark had a higher proportion of both high dose exposures and severe CHD.

**Table 4:** High Dose exposure^a^ and ‘all anomalies’, CHD, severe CHD, ‘Stillbirth or Anomaly’: 3 countries.

	High dose LMP±91 days n = 1429		Other dose LMP±91 days n = 11,533		Unexposed LMP±91 days n = 506,155		Meta regression^b^
	N	% of exposed	N	% of exposed	N	% of exposed	OR (95%CI)
Anomaly or stillbirth	53	3.71	420	3.64	15,829	3.13	**1.10 (1.02–1.20)**
All anomalies	43	3.01	357	3.10	13,525	2.67	1.08 (0.99–1.17)
CHD	18	1.26	103	0.89	4495	0.89	1.06 (0.91–1.24)
Severe CHD	7	0.49	27	0.23	864	0.17	**1.49 (1.13–1.97)**

^a^Exclusions and exposures as Table 1.

^b^No measure of heterogeneity is available. ORs quoted represent category increases in dose.

### Confounding by co-exposure

Adjusting for smoking and SES made little difference to ORs. Adjusting for smoking reduced the numbers of exposed cases, due to missing data disproportionately affecting the cases (Table Aa in S1 Appendix) and uncertainty over ex-smokers, and hence widened confidence intervals (Table 5). Checks indicated that: 52 of the 400 exposed cases had been exposed to prescription medicines identified as potentially teratogenic (listed under ‘confounding’), benzodiazepines (21) thyroxine (13), antipsychotics first generation (9), second generation (5), angiotensin converting enzyme inhibitors (3), lithium (1), and 0 for all other exposures; 30 were exposed to maternal ill-health; 14 had siblings in the dataset with anomalies, and 12 had mothers with an anomaly recorded. Of the 34 exposed severe CHD cases, 6 were also exposed to potential teratogens of varying potency, benzodiazepines (2 or <5), thyroxine (2), lithium (1), and first generation antipsychotics (1), 2 were exposed to maternal ill-health and none had maternal siblings or mothers with any congenital anomaly.

**Table 5:** Congenital anomalies and stillbirths and SSRI exposure LMP±91 days^a^: analyses adjusted for smoking and socio-economic status (SES).

	Adjusted analysis		Unadjusted analysis		Number of exposed cases	
	Meta OR (95% CI)	I^2^	Meta OR (95% CI)	I^2^	adjusted analysis	Un-adjusted analysis^c^
Outcome adjusted for smoking						
All Anomalies	1.08 (0.97–1.20)	0	1.09 (0.99–1.21)	0	366	400
CHD	1.00 (0.82–1.21)	46.4	1.03 (0.86–1.24)	55.66	108	121
Severe CHD	1.43 (0.99–2.07)	47.9	**1.50 (1.06–2.11)**	51.36	30	34
Anomaly or stillbirth	**1.12 (1.01–1.23)**	0	**1.13 (1.03–1.24)**	0	433	473
Outcome adjusted for SES^b^: lowest vs. the rest						
All Anomalies	1.09 (0.98–1.21)	0	1.09 (0.99–1.21)	0	398	400
CHD	1.03 (0.85–1.23)	25.1	1.03 (0.86–1.24)	55.66	120	121
Severe CHD	**1.47 (1.04–2.08)**	23.6	**1.50 (1.06–2.11)**	51.36	34	34
Anomaly or stillbirth	**1.12 (1.02–1.23)**	0	**1.13 (1.03–1.24)**	0	471	473

^a^Exclusions and exposures as Table 1.

^b^SES: years in education in Denmark and Norway and Townsend fifth in Wales (Tables Aa-c in S1 Appendix).

When SES was examined as a linear trend, results were essentially unchanged. The decision to compare the most deprived with the rest was based on data in Tables E, F in S1 Appendix.

Numbers were too low for adjusted analyses in some anomalies of interest, including abdominal wall defects. In Wales, abdominal wall defects and SSRI exposure were associated with smoking and SES (Table F in S1 Appendix).

^c^Unadjusted analyses are reproduced here for the convenience of readers.

### Confounding by indication

#### a) Exposure patterns

Including only women present in the database from 1 year before to 1 year after pregnancy reduced the population (Fig 1), but left prevalence of anomalies and SSRI exposure largely unchanged. Prevalence appeared lowest in those never exposed, increasing in stoppers, pausers and those exposed during pregnancy, but differences were not statistically significant. The prevalence of severe CHD appeared lower (0.23%) in those prescribed ≥1 SSRI prescription(s) in the 91 days either side of LMP than those receiving >1 prescription (0.31%) (Table 6 and Table G in S1 Appendix).

**Table 6:** Comparisons of stopping before pregnancy, pausing during pregnancy, exposure LMP±91 days^a^, and unexposed for 11 quarters^b^ for all anomalies, CHD and severe CHD, including receipt of >0 and >1 prescriptions: 3 countries.

**Exposed >0 SSRI prescription n = 11,512**								
	Unexposed 11 quarters^b^		Stoppers		Pausers		Exposed LMP±91 days	
	N	%	N	%	N	%	n	%
Total	426,962		6315		2203		11,512	
All anomalies	11,049	2.59	175	2.77	62	2.81	341	2.96
CHD	3651	0.82	64	1.01	24	1.09	94	0.82
SevereCHD	722	0.16	9	0.14	4	0.18	26	0.23
**Exposed >1 SSRI prescription n = 6392**								
	Unexposed 11 quarters^b^		Stoppers		Pausers		Exposed LMP±91 days	
	N	%	N	%	N	%	n	%
Total	426,962		3146		923		6392	
All anomalies	11,049	2.59	87	2.77	26	2.82	190	2.97
CHD	3651	0.82	29	0.92	11	1.19	56	0.88
Severe CHD	722	0.16	6	0.19	3	0.33	20	0.31

^a^Exclusions as Table 1 plus ‘not on database for 1 year either side of pregnancy’.

^b^11 quarters—pregnancy and 1 year either side.

A full version of this table, with ORs and 95% CIs is available in Table G in S1 Appendix. For all anomalies and severe CHD, differences between exposed to >1 SSRI prescription and unexposed yielded 95% confidence intervals excluding one.

#### b) Depression in Wales

For women present on the database during pregnancy and 1 year either side (n = 103,204), a recorded diagnosis of depression (ever) was not associated with increased prevalence of all anomalies (OR 1.01, 0.91–1.12, Table 7 and Table H in S1 Appendix). The prevalence of anomalies amongst those with a diagnosis of depression, medicated (3.22%) or unmedicated (3.19%) (Table H in S1 Appendix), was slightly lower than following SSRI exposure, regardless of diagnosis (3.44%) (Table 1), but such differences were not statistically significant. More infants exposed to medicated than unmedicated depression had severe CHD (OR 1.60, 0.70–3.66), but numbers were low (Table 7 and Table H in S1 Appendix).

**Table 7:** Depression, medicated and unmedicated and congenital anomalies and stillbirths in Wales.^a^

	depression diagnosed (ever) n = 13189		no depression recorded n = 90015			Depression exposed to N06AB LMP±91 days n = 2897		Depression un-medicated with N06AB LMP±91 days n = 10292		
	N	% of diagnosed depressed	n	% of no depression recorded	OR (95% CI)	N	% of exposed	N	% of un-medicated	OR (95% CI)
Population	13,189	100	90,015	100		2897	100	10,292	100	
Anomaly or stillbirth	486	3.68	3158	3.51	1.05 (0.96–1.16)	108	3.75	378	3.67	1.00 (0.82–1.26)
aL1 All anomalies	422	3.20	2844	3.16	1.01 (0.91–1.12)	93	3.21	329	3.20	1.00 (0.79–1.27)
aL3 Neural tube	10	0.08	97	0.11	0.70 (0.37–1.35)	<5		6–9		>1
aL17CHD	141	1.07	837	0.93	1.15 (0.96–1.38)	23	0.79	118	1.15	0.69 (0.44–1.08)
aL97Severe CHD	24	0.18	172	0.19	0.95 (0.62–1.46)	7	0.24	17	0.17	1.46 (0.61–3.53)
aL49 Abdo wall defects	16	0.12	60	0.07	**1.82 (1.05–3.16)**	<5		12–15		>1
al50 Gastroschisis	11	0.08	37	0.04	**2.03 (1.04–3.98)**	<5		7–10		>1
aL54 Renal dysplasia	9	0.07	68	0.08	0.90 (0.45–1.81)	<5		5–8		>1
aL59 Hypospadias	34	0.26	246	0.27	0.94 (0.66–1.35)	<5		30–33		>1
aL 61 Limb reduction	7	0.05	64	0.07	0.75 (0.34–1.64)	0		7	0.10	NA
aL66 Talipes equinovarus	23	0.17	158	0.17	0.99 (0.64–1.51)	<5		19–22		<1
al 101: Oro-facial clefts	17	0.12	136	0.15	0.85 (0.52–1.41)	6	0.21	11	0.11	1.94 (0.72–5.25)

^a^Exclusions and exposures as Table 1 plus ‘not on database for 1 year either side of pregnancy’. Increased time on database was associated with a diagnosis of depression and increased deprivation, but not congenital anomalies, and correlation with maternal age was low (r = -0.06).

Values for any antidepressant (N06A) exposure are presented in Table H in S1 Appendix.

NA—unable to calculate. N06AB—any SSRI.

Deprivation was associated with depression and SSRI prescription (Table E in S1 Appendix). We found no significant associations between all anomalies, ‘anomalies and stillbirths’, CHD, severe CHD and: smoking, substance misuse or heavy drinking, antipsychotics or deprivation. Abdominal wall defects were associated with deprivation and smoking (Table F in S1 Appendix).

In subgroups of women in Wales recorded (ever) as heavy drinkers or substance misusers, the most deprived fifth, those prescribed antipsychotics within 91 days of LMP, and smokers, additional SSRI exposure appeared to increase the prevalence of congenital anomalies (Table 8).

**Table 8:** Subgroup explorations in Wales: SSRI exposure and congenital anomalies or Stillbirths.^a^

	SSRI exposure 91 days either side of LMP		
	SSRI exposed LMP±91 days n (% exposed)	Not SSRI exposed LMP±91 days n (% not exposed)	OR (95%CI) where available
Heavy drinking or substance misuse recorded (n = 1658)			
Number	288	1370	
All Anomalies	18 (6.3)	38 (2.8)	**2.34 (1.31–4.16)**
CHD	6 (2.1)	13 (0.9)	2.22 (0.85–5.89)
Severe CHD	<5	<5	>1 (P>0.05)
Anomaly or stillbirth	19–22 (6.6–7.6)	44 (3.2)	**>1 (<0.05)**
Most deprived fifth (Townsend index of material deprivation) (n = 25,763)			
Number	1910	23,853	
All Anomalies	70 (3.7)	781 (3.3)	1.12 (0.88–1.44)
CHD	19 (1.8)	235 (1.0)	1.01 (0.63–1.62)
Severe CHD	5 (0.3)	47 (0.2)	1.33 (0.53–3.35)
Anomaly or stillbirth	75 (3.9)	870 (3.6)	1.08 (0.85–1.37)
Exposed to any antipsychotic^b^ at any time (n = 833)			
Number	266	567	
All Anomalies	9/266 (3.4)	16/567 (2.8)	1.21 (0.53–2.77)
CHD	<5		<1 P >0.05
Severe CHD	<5		>1 P >0.05
Anomaly or stillbirth	10–13 (3.8–4.9)	17–21 (3.0–3.7)	>1 P >0.05
Smokers^c^ (n = 30,534)			
Number	2583	27,951	
All Anomalies	92/2583 (3.6)	904/27,951 (3.2)	1.11 (0.89–1.38)
CHD	23/2583 (0.89)	265/27,951 (0.9)	0.94 (0.61–1.44)
Severe CHD	7/2583 (0.27)	49/27,951 (0.2)	1.55 (0.70–3.42)
Anomaly or stillbirth	110/2583 (4.3)	1019/27,951 (3.6)	1.18 (0.96–1.44)

^a^Exclusions and exposures as Table 7.

^b^For antipsychotic and benzodiazepine exposure see Table F in S1 Appendix.

^c^Although smoking was well recorded, some 15% women were classified as ex-smokers, with no cessation date; fieldwork experience indicates that some women self-report their smoking status as ‘ex’ when discontinuation has been <24 hours.

Amongst the 110 live birth cases of Down syndrome, exposure to SSRIs increased the incidence of CHD from 60/101 (60%) to 9/9 (100%) (RR 1.68, 1.43–1.98).

Abdominal wall defects: too few cases to report.

Recorded recreational drug use was implausibly low, and not analysed.

## Discussion

Congenital anomalies appeared more prevalent amongst infants exposed than unexposed to prescription of SSRIs within 91 days of 1^st^ day of LMP, consistent with recent meta-analyses[26,30,73]; this difference was not statistically significant. However, the increase was significant for the composite adverse outcome of ‘anomaly or stillbirth’ (OR 1.13, 1.03–1.24, NNH 192). Significant dose-response relationships were found between SSRI prescription and ‘anomaly or stillbirth’ and severe CHD (meta-regression ORs 1.10, 1.02–1.20, and 1.49, 1.12–1.97), supporting work on paroxetine[34] and umbilical cord samples[74], but contrary to reports with fewer exposed cases[15], and different classifications[32].

The literature’s inconsistency regarding SSRIs and CHD is reflected in our incongruent findings for all CHD and severe CHD. The dose-response association between SSRI prescription and severe CHD (Table 4) appears stronger than for all CHD, but there is insufficient power to test this hypothesis. The association with severe CHD supports some studies[15,30,31], while the absence of association with all CHD reflects others[22,29,32,33], suggesting that SSRIs may only affect certain cardiac anomalies. As elsewhere, paroxetine was associated with all CHD and VSD[26,31,33], possibly attributable to its saturation kinetics[47]. Some previously reported associations were confined to a single SSRI: neural tube defects [33,35] with fluoxetine (OR 2.57, 1.21–5.46), escitalopram with talipes equinovarus[52], citalopram with hypospadias [27,75] (Table C in S1 Appendix). Genetic variation[76] and induction of the cytochrome P450 system, essential for SSRI metabolism, which occurs early in pregnancy, may reduce SSRI bioavailability and mitigate any adverse impact[51,77], particularly at standard doses.

Depression and social stressors are associated with activation of the hypothalamic-pituitary-adrenal (HPA) axis, pro-inflammatory cytokines[78], and placental equivalents[79], which affect organogenesis[80], foetal growth[81], and birth outcome[82]. We found no association between anomalies linked with maternal social stressors (oro-facial clefts)[83] and SSRIs, antidepressants or depression (Table 7 and Tables C, H in S1 Appendix). This intimates that independent serotoninergic[11] and vasoconstrictor[9,50] mechanisms might underlie adverse outcomes following SSRI exposure [84]. SSRI-induced vasoconstriction[9,51] may explain associations between SSRIs and low birth weight, growth restriction[85–87], and persistent pulmonary hypertension[88], contributing to synergy in our composite adverse outcome. (Growth restriction accounts for 43% of stillbirths [89]). Stillbirth is a relatively rare outcome (prevalence <0.5%), not previously associated with SSRI exposure[90,91].

Adjusting for smoking and SES left findings largely unchanged (Table 5), as elsewhere[16,28]. Exploration in Wales found no evidence for major confounding, except for abdominal wall defects (Table F in S1 Appendix). Excluding subjects exposed to insulin, AEDs and coumarins reduced the need to adjust for co-exposure[32]. SSRI prescription conferred additional risks on those co-exposed to substance misuse or heavy drinking or other psychoactive medicines[17,92–95] (Table 8).

To disentangle SSRI exposure from depression, like others [15], we compared those exposed to SSRIs with those where prescriptions had been stopped or paused. Although prevalence of ‘all anomalies’ and severe CHD was lower in those who stopped rather than continued prescriptions, confidence intervals were wide, indicating limited power of this analysis and the presence of confounding (Table 6 and Table G in S1 Appendix). In Wales, we analysed ‘any record of depression’, based on practitioners’ reluctance to repeat data entries and the ‘depression diathesis model’, which suggests that any episode may predispose to stressor-induced release of pro-inflammatory cytokines, permanently altering hippocampal, prefrontal and frontocingulate neurochemistry and connections[78]. We found no associations between depression and anomalies (Table 7 and Table H in S1 Appendix), supporting suggestions that depression and antidepressants may act separately[84,96] in modifying release of pro-inflammatory cytokines that affect organogenesis. Similarly, meta-analysis indicates that increased risks of preterm birth persist when SSRI exposed are compared with unmedicated controls diagnosed with depression [97]. Our definition of depression (any record, ever) may contribute to incongruence with other reports[22,98]. We acknowledge that prescription or resumption or higher doses of SSRIs may indicate on-going, recurrent or more severe depression, compounding the difficulties of disentangling the effects of prescriptions from underlying illness.

### Strengths and limitations

Findings are strengthened by: precise diagnostic coding of congenital anomalies [37]; inclusion of TOPFA cases and stillbirths; contemporary controls; accounting for exposure to other antidepressants and SES; prospective data[99], free from recall bias [100]: these may explain differences with the published literature [15,16,24,32,33]. Most infants exposed to SSRIs in early pregnancy were not exposed in late pregnancy [3], reducing any over-ascertainment of anomalies in neonatal assessments of ‘high risk’ infants: the main concern is conflation of ASD with patent foramen ovale, which is precluded by EUROCAT coding [37]. Where associations were observed, effects were modest (ORs below 2), and the low numbers of exposed cases necessitate cautious interpretation, but the associations with severe CHD and ‘major anomaly or stillbirth’ are strengthened by dose-response relationships[101]. **Generalization** of findings on ‘all anomalies’, with or without stillbirths is strengthened by consistency across different populations (I^2^ = 0 for both analyses) (Table 3 and Table C in S1 Appendix). Our findings are limited by: dilution of exposure; incomplete recording in electronic databases; and study size.

Our **effect sizes** may be conservative and ORs diluted by our extended exposure window, and threats to prescription adherence. Our extended time window before LMP (91 days) was based on typical prescription duration and pharmacokinetic parameters, which differ between SSRIs. We acknowledge that this may have led to some unexposed subjects being misclassified as exposed, diluting ORs [102], particularly for SSRIs with shorter half-lives (citalopram, sertraline, escitalopram, and low dose paroxetine)[47], possibly explaining divergent findings[16,32,103].

**Adherence to prescribed regimens** cannot be ascertained from prescription or dispensing data. However, it is more likely where >1 prescription is issued, and therefore our stronger findings when analysing women exposed to >1 prescription are consistent with dilution due to exposure misclassification from non-adherence. International differences in exposure observed may, in part, reflect differences in issued (Wales) versus redeemed (Norway, Denmark) prescriptions. Prescription non-redemption varies between settings; reviewers suggest a mean of 16.4% (range 11–19%)[104]. For antidepressants, estimates range from 20% in the USA where affordability is a prominent concern[105], to 4% in the Netherlands [106] and 4.5% (CNS medicines) in UK primary care [107].

**Electronic cohorts** based on prospectively collected routine data facilitate pharmacovigilance across whole populations; however, clinical details (other than EUROCAT coding) including indications for prescriptions and severity of illness, confounding variables and genotype may be incompletely recorded. Dose-response explorations were based on tablet size, and we were unable to take account of formulation or number of tablets or packets prescribed. Genetic vulnerability to environmental factors, including SSRIs, is hypothesised [76], but rarely recorded. Some anomalies, including some not associated with recognised syndromes, can result from inherited conditions[108]. However, family histories tend to be incompletely recorded, and there may be no information on fathers and other family members, including any paternal half-siblings. Information on paternity is also difficult to obtain in fieldwork[109]. CHDs are associated with maternal CHD[65], and even a small number of affected women might affect our interpretation of the severe CHD outcome. Although we checked as thoroughly as possible, data on maternal morbidities were limited by timeframes of databases and, possibly, incomplete recording. We did not exclude women with: diabetes not prescribed insulin, unmedicated epilepsy, glucose-6-phosphate dehydrogenase deficiency, sickle cell anaemia, maternal hypertension. BMI was poorly recorded in all databases, precluding exploration of confounding by obesity[110].

**Recreational drug use, heavy alcohol use and substance misuse** are captured poorly in clinical care, fieldwork and databases. These potential confounders were not available in the Scandinavian databases. Only problems recorded by primary care professionals could be identified in Wales; this would not include casual users or regular users not reporting problems. Depression may be under-reported in primary care records, due to inaccurate diagnosis by primary care practitioners[111], fears of ‘labeling’ or stigmatizing[112], and, possibly, incomplete record transfer from secondary care. Accordingly, we acknowledge the risks of under-ascertainment, and the limitations of taking the absence of any records as indicative of non-exposure. Adjustment was limited by low numbers of exposed cases, incomplete recording of smoking (Table 6 and Tables Aa-c, G in S1 Appendix) and, in Denmark, a higher prevalence of missing data amongst cases (Tables Aa-c in S1 Appendix). However, in Wales, alternative predictors were not identified for anomalies other than abdominal wall defects (Table F in S1 Appendix).

Analyses of all antidepressants and SSRIs excluding co-prescription of other antidepressants could not include Denmark, and therefore are not directly comparable to the main results.

**The study’s size** was sufficient (>312,000) to detect an association between SSRI exposure (2.50%) with major anomalies (prevalence 2.68%, Table 1) greater than OR 1.2, with 80% power and alpha 0.05, but >1,000,000 subjects would be needed to detect ORs of 1.1. For the commonest anomaly, CHD (prevalence 0.9%) and the commonest SSRI (citalopram, 0.8% exposure), there were sufficient subjects (>456,000) to detect an OR of 1.5 [113]. Higher prescription rates in Wales gave more exposed pregnancies (and power) than previous cohorts[14,21] benefitting from verified EUROCAT coding[15,22,33].

We acknowledge the hazards of **multiple testing**, without correction, but recognise the tensions between umbrella terms, which can hide true signals between specific anomalies and specific medicines, and narrow categories or rare outcomes yielding numbers too small for statistical comparisons[114,115]. *A priori* hypotheses[9,27,31,35,51] were tested without statistical adjustment, to limit misinterpretation (Table 3)[116]. Associations between individual agents and anomalies offer signals for replication in independent data sets (Table C in S1 Appendix). Our population-based cohort study yielded lower ORs than Wemakor *et al’s* [31] case-malformed control study of 12 EUROCAT registries, suggesting that we have not over-estimated harms, congruent with reports that estimates of adverse event rates are lower in cohort than case-control studies[117].

Logical and biological inferences should be considered when interpreting these findings, which are congruent with seven of the nine Bradford-Hill criteria of causation [118]: temporal and dose-response relationships; consistency of effect size (ORs) for ‘all anomalies’ internally, and with the literature[26,30,73]; biological plausibility; consideration of alternative explanations (depression, SES, smoking); specificity to severe CHD; and coherence with extant theories of serotonergic transmission and vasoconstriction. However, neither we nor others offer experimental evidence, and the associations, while persistent and clinically serious [60], represent small absolute risk differences (Table 3). Where partial overlap between our data and that published by EUROCAT[31], the Danish 1995–2008[103], and Nordic authors 1996–2010[16] occurs, findings are consistent. However, the last excluded stillbirths and TOPFAs, reducing the prevalence of anomalies. We have avoided P values, but acknowledge the problems inherent in dichotomizing data according to 95% confidence intervals where assignment is not randomized and assumptions (for example on adherence) are unverifiable [119]: interpretation and translation into clinical practice rest with readers.

### Interpretation and care pathways

Our findings resonate with larger cohorts and meta-analyses[26],[30],[73], despite the risks of attenuation of odds ratios, above. SSRI prescription in the 91 days either side of LMP was associated with higher prevalence of anomalies and stillbirths: ~7 rather than ~6 adverse outcomes per 200 exposed births. The heterogeneity in severe CHD may be attributable to diverse prescription regimens, environmental factors or ill-defined contextual variables. The higher prevalence was most apparent in Denmark, where single prescriptions were unusual and high doses relatively common, and absent in Norway, where single prescriptions were more common, high doses and paroxetine relatively rare, and escitalopram the most popular SSRI. The UK formulary [120] notes the pro-arrhythmic potential of escitalopram and contra-indicates breastfeeding, which may explain lower use in Wales.

Antidepressant use in pregnancy is determined by the balance between benefits to the woman and harms to the foetus: there is no certainty that realisation of the fourth (reducing child mortality) and fifth (improving maternal health) UN Millennium Development goals[121] will always coincide. Congenital anomalies and stillbirths are not the only harms associated with antidepressants[122]. Prescribing decisions are informed by other possible harms[73,123]: spontaneous abortion[29,124], low birth weight, prematurity, admission to neonatal special care facilities [33,85–87,125], gestational hypertension, postpartum haemorrhage[126], persistent pulmonary hypertension in neonates[88,120,127,128], concerns over delayed motor development[129].

The uncertain clinical effectiveness of antidepressants in pregnancy[126,130] for mild or moderate depression[131,132], treatment resistance in some 30% patients[78], contra-indication in mild depression [120], and wide variations in prescribing across Europe[3] underlie recommendations to restrict pharmacotherapy in pregnancy to women with severe depression[133–135] or adjust prescribing thresholds[73]. However, poor parental perinatal mental health can adversely affect childhood outcomes [136], while effects on perinatal outcomes are modest [137], and more marked in developing countries [138]. Guidelines’ equivocation places the onus on prescribers [139,140], despite constraints in primary care, including short appointment times[141]. Withholding or withdrawing antidepressants from women with serious illness may worsen illness or induce withdrawal symptoms or relapse[133,142], and clinicians are mindful of the increased risk of harm, including suicide, in the 28 days following discontinuation[143], but more evidence relating to those less seriously ill is needed. The higher exposure rates in Wales, compared with Norway and Denmark, plus the low prevalence of mental health diagnoses [66], may suggest that women in Wales suffering less severe depression are more likely to be prescribed SSRIs. These women may derive less benefit from antidepressants, whilst risking the same harms.

### Implications

The clinical importance of stillbirth and major congenital anomalies, including severe CHD, suggests that small increases in absolute risk might influence decisions on therapy and care pathways at population level[144]. Examination of three Northern European population cohorts consistently indicated an association between SSRIs and major anomalies, which increased when stillbirths were included. Uniquely, the association identified with severe CHD was supported by: a dose-response relationship, lower prevalence in those stopping SSRIs, higher prevalence in those with >1 prescription, minimal confounding by SES, plus, in Wales, no association with alternative exposures, including depression. Given the rarity of specific congenital anomalies and ethical considerations, randomised trials with these outcomes may never be undertaken. However, since risk estimates for adverse events are similar in trials and observational studies[145], these findings have implications for practice.

Even if associations reported here are not necessarily causal, SSRI prescriptions can be identified in primary care records and offer convenient markers for increased vulnerability, more easily ascertained and reliable than smoking status or recreational drug consumption [109,146]. Balancing the number needed to harm, 192, with the severity of potential adverse effects (stillbirth or major anomaly) [53], whilst minimising any iatrogenic harm [140] might entail regarding records of SSRI prescriptions as indication to:

- Target all women contacting primary care for SSRI prescriptions, not just those identifying themselves as planning pregnancy, since ~43% UK pregnancies are unplanned[147].
- Regard substance misuse or heavy drinking as possible indicators of high risk from SSRI prescribing (6.3%).
- Expand pre-conception care to include: reviewing therapeutic regimens, *particularly high doses of SSRIs*; reflecting that ~40% women discontinuing SSRIs after conception do not restart within a year of childbirth [3], and cognitive behavioural therapy may be equally effective[148]; prescribing folic acid, which may reduce the prevalence of CHD[149].
  - Consider offering women prescribed SSRIs in pregnancy third trimester scans or alternative continuous monitoring technology to:
    - take advantage of advances in monitoring and surgery *in utero*
    - ensure appropriate levels of neonatal care are available at birth.
  - Consider whether there is now sufficient evidence and clinical indication to offer a modified care pathway to include detailed ultrasound scans with views of the 4 cardiac chambers, outflow tracts and aortic arch plus Doppler investigation of blood flow [150], even if not otherwise indicated. Ultrasound is not considered to be associated with risk, and there are no reported harms [151], with follow up to age 15–16 [152]; some may consider that the injunction “Do no harm” [140] might justify the additional clinical work, and any additional anxiety for parents associated with clinically unimportant incidental findings.

## Supporting Information

S1 Appendix Supplementary tables. **Tables Aa-c**. The populations. **Tables Ba and Bb**. Anomalies and exposures for each SSRI and all antidepressants. **Table C**. Anomalies and SSRI exposure for each agent with data from 3 countries. **Table D**. Anomalies and SSRI exposure with and without antidepressants. **Table E**. Deprivation and selected exposures in Wales. **Table F**. Exploration of anomalies and alternative exposures in Wales. **Table G**. Comparisons of stopping before pregnancy, pausing during pregnancy, exposure LMP±91 days, and unexposed for 11 quarters for all anomalies, CHD and severe CHD, including receipt of >0 and >1 prescriptions: 3 countries. **Table H**. Depression, medicated and unmedicated and congenital anomalies and stillbirths in Wales.
(DOCX)

S2 Appendix STROBE statement. (DOCX)
