# Supplementary material for: Selective Serotonin Reuptake Inhibitor (SSRI) Antidepressants in Pregnancy and Congenital Anomalies: Analysis of Linked Databases in Wales, Norway and Funen, Denmark
Source: PLoS One. 2016 Dec 1;11(12):e0165122. doi: 10.1371/journal.pone.0165122 (PMC5131901; doi:10.1371/journal.pone.0165122)
Supplement: S1 Appendix — Tables Aa-c. The populations. Tables Ba and Bb. Anomalies and exposures for each SSRI and all antidepressants. Table C. Anomalies and SSRI exposure for each agent with data from 3 countries. Table D. Anomalies and SSRI exposure with and without antidepressants. Table E. Deprivation and selected exposures in Wales. Table F. Exploration of anomalies and alternative exposures in Wales. Table G. Comparisons of stopping before pregnancy, pausing during pregnancy, exposure LMP±91 days, and unexposed for 11 quarters for all anomalies, CHD and severe CHD, including receipt of >0 and >1 prescriptions: 3 countries. Table H. Depression, medicated and unmedicated and congenital anomalies and stillbirths in Wales. (DOCX) [file pone.0165122.s001.docx]

# S1 Appendix: supplementary tables

Tables Aa-Ac the populations

Tables Ba and Bb Anomalies and exposures for each SSRI and all antidepressants

Table C. anomalies and SSRI exposure for each agent with data from 3 countries

Table D. anomalies and SSRI exposure with and without antidepressants

Table E. Deprivation and selected exposures in Wales

Table F. Exploration of anomalies and alternative exposures in Wales

Table G. Comparisons of stopping before pregnancy, pausing during pregnancy, exposure LMP±91 days, and unexposed for 11 quarters for all anomalies, CHD and severe CHD, including receipt of >0 and >1 prescriptions: 3 countries

TableH. Depression, medicated and unmedicated and congenital anomalies and stillbirths in Wales^a^

#### Table A. Demographic Details of Populations.

#### Aa. Denmark. total population 58,234

|  | exposed to any SSRI LMP±91 days n=1335 | | | | not exposed n=56899 | | | |
| --- | --- | --- | --- | --- | --- | --- | --- | --- |
|  | cases/ any anomaly | | not cases (no anomaly) | | cases/ any anomaly | | not cases |  |
|  | n | % | n | % | n | % | n | % |
| Number of pregnancy outcomes | 42 | 3.15 | 1293 | 96.85 | 1651 | 2.90 | 55248 | 97.10 |
| Distinct number of females | 41 | 3.44 | 1151 | 96.56 | 1598 | 4.17 | 36744 | 95.83 |
| Distinct number of pregnancies | 42 | 3.19 | 1274 | 96.81 | 1644 | 2.95 | 54008 | 97.05 |
| number of infants with a chromosomal, genetic, teratogenic anomaly (as in exclusions) | 7 | 16.67 | 0 | 0.00 | 339 | 20.53 | 0 | 0.00 |
| number of infants exposed to insulin in pre1 and tri1 | 0 | 0.00 | 17 | 1.31 | 27 | 1.64 | 498 | 30.16 |
| number of infants exposed to AEDs pre4 to post4 | <5 |  | 22 | 1.70 | 11 | 0.67 | 207 | 12.54 |
| number of infants exposed to coumarins pre1 and tri1 | no data |  |  |  |  |  |  |  |
| Outcome |  |  |  |  |  |  |  |  |
| - Live birth | 32 | 76.19 | 1284 | 99.30 | 1348 | 81.65 | 54898 | 3325.14 |
| - Stillborn | 0 | 0.00 | 9 | 0.70 | 33 | 2.00 | 350 | 0.63 |
| - TOPFA | 10 | 23.81 | 0 | 0.00 | 270 | 16.35 | 0 | 0.00 |
| Mean age at pregnancy end [years: (SD)] | 29 [6.06] | | 28.95 [5.39] | | 29.79 [5.19] | | 29.07 [4.8] | |
| Socioeconomic status |  |  |  |  |  |  |  |  |
| - Further education | 11 | 26.19 | 397 | 30.70 | 693 | 41.97 | 22971 | 41.58 |
| - Vocational training | 16 | 38.10 | 414 | 32.02 | 548 | 33.19 | 18850 | 34.12 |
| - 12 years | <5 |  | 89 | 6.88 | 93 | 5.63 | 3223 | 5.83 |
| - 10 years or less | 13 | 30.95 | 377 | 29.16 | 300 | 18.17 | 9410 | 17.03 |
| - Further education |  |  |  |  |  |  |  |  |
| - unknown | 0 | 0.00 | 16 | 1.20 | 17 | 1.03 | 794 | 1.44 |
| Smoking status |  |  |  |  |  |  |  |  |
| - non-smoker | 22 | 52.38 | 894 | 69.14 | 1106 | 66.99 | 44436 | 80.43 |
| - current smoker | 10 | 23.81 | 373 | 28.85 | 231 | 13.99 | 9194 | 16.64 |
| - ex-smoker | NA | NA | NA | NA | NA | NA | NA | NA |
| - unknown | 10 | 23.81 | 26 | 2.01 | 314 | 19.02 | 1618 | 2.93 |

#### Ab Norway. total population 351,825

|  | exposed to any SSRI LMP±91 days n=5790 | | | | not exposed n=346035 | | | |
| --- | --- | --- | --- | --- | --- | --- | --- | --- |
|  | cases/ any anomaly | | not cases |  | cases/ any anomaly | | not cases |  |
|  | n | % | n | % | n | % | n | % |
| Number of pregnancy outcomes | 178 | 3.07 | 5612 | 96.93 | 10134 | 2.9 | 335901 | 97.1 |
| Distinct number of females | 155 | 3.29 | 4559 | 96.71 | 8013 | 2.32 | 338022 | 97.68 |
| Distinct number of pregnancies | 177 | 3.12 | 5505 | 96.88 | 10056 | 2.96 | 329911 | 97.04 |
| number of infants with a chromosomal, genetic, teratogenic anomaly (as in exclusions) | 19 | 10.67 | 0 | 0.0 | 1272 | 12.55 | 0 | 0.0 |
| number of infants exposed to insulin pre1 and tri1 | 5 | 2.81 | 62 | 1.10 | 110 | 1.09 | 1526 | 0.45 |
| number of infants exposed to AEDs pre4 to post4 | 7 | 3.93 | 247 | 4.40 | 77 | 0.76 | 1691 | 0.50 |
| number of infants exposed to coumarins pre1 and tri1 | <5 |  | <5 |  | 8 | 0.08 | 107 | 0.03 |
| Outcome |  |  |  |  |  |  |  |  |
| - Live birth | 158 | 88.76 | 5568 | 99.22 | 8914 | 87.96 | 334119 | 99.47 |
| - Stillborn | <5 |  | 38 | 0.68 | 119 | 1.17 | 1614 | 0.48 |
| - TOPFA | 18 | 10.11 | 6 | 0.11 | 1101 | 10.86 | 168 | 0.05 |
| Mean age at pregnancy end [years: (SD)] | 29.3 [5.88] | | 29.5 [5.5] | | 30.3 [5.3] | | 29.7 [5.1] | |
| Socioeconomic status |  |  |  |  |  |  |  |  |
| - high >12 years of education | 47 | 26.40 | 1912 | 34.07 | 5090 | 50.23 | 168564 | 50.18 |
| - medium 10-12 years of education | 69 | 38.76 | 1933 | 34.44 | 2962 | 29.23 | 99611 | 29.65 |
| - low 0-9 years of education | 60 | 33.71 | 1693 | 30.17 | 1754 | 17.31 | 56926 | 16.95 |
| Unknown | <5 |  | 74 | 1.32 | 328 | 3.24 | 10800 | 3.22 |
| Smoking status |  |  |  |  |  |  |  |  |
| - non-smoker | 94 | 52.81 | 3203 | 57.07 | 6940 | 68.48 | 240064 | 71.47 |
| - current smoker | 54 | 30.34 | 1570 | 27.98 | 1124 | 11.09 | 38441 | 11.44 |
| - ex-smoker | NA |  | NA |  | NA |  | NA |  |
| - unknown | 30 | 16.85 | 839 | 14.95 | 2070 | 20.43 | 57396 | 17.09 |

#### Ac Wales. Total population 117,717

|  | exposed to any SSRI LMP±91 days n=6514 | | | | not exposed n=111203 | | | |
| --- | --- | --- | --- | --- | --- | --- | --- | --- |
|  | cases/ any anomaly | | not cases |  | cases/ any anomaly | | not cases |  |
|  | n | % | n | % | n | % | n | % |
| Number of pregnancy outcomes | 260 | 3.99 | 6254 | 96.01 | 4118 | 3.70 | 107085 | 96.30 |
| Distinct number of females | 169 | 3.81 | 4268 | 96.19 | 2624 | 3.36 | 75394 | 96.64 |
| Distinct number of pregnancies | 255 | 3.98 | 6152 | 96.02 | 4031 | 3.68 | 105433 | 96.32 |
| number of infants with a chromosomal, genetic, teratogenic anomaly (as in exclusions) | 36 | 13.85 | 0 | 0.00 | 618 | 15.01 | 0 | 0.00 |
| number of infants exposed to insulin in pre1 and tri1 | <5 |  | 36 | 0.58 | 31 | 0.75 | 331 | 0.31 |
| number of infants exposed to AEDs pre4 to post4 | 10 | 3.85 | 147 | 2.35 | 54 | 1.31 | 1017 | 0.95 |
| number of infants exposed to coumarins in pre1 and tri1 | <5 |  | 5 | 0.08 | <5 |  | 43 | 0.04 |
| Outcome |  |  |  |  |  |  |  |  |
| - Live birth | 221 | 85.00 | 6222 | 99.49 | 3568 | 86.64 | 106663 | 99.61 |
| - Stillborn | <5 |  | 32 | 0.51 | 29 | 0.70 | 399 | 0.37 |
| - TOPFA | 35-38 |  | 0 | 0.00 | 521 | 12.65 | 23 | 0.02 |
| Mean age at pregnancy end [years: (SD)] | 28.98 [6.35] | | 28.04 [5.97] | | 28.63 [6.36] | | 28.23 [6.07] | |
| Socioeconomic status (Townsend et al 1988) |  |  |  |  |  |  |  |  |
| - Wales Townsend fifth = 1 (least deprived) | 35 | 13.46 | 755 | 12.07 | 794 | 19.28 | 18978 | 17.72 |
| - 2 | 31 | 11.92 | 954 | 15.25 | 717 | 17.41 | 20697 | 19.33 |
| - 3 | 52 | 20.00 | 1194 | 19.09 | 778 | 18.89 | 20892 | 19.51 |
| - 4 | 56 | 21.54 | 1464 | 23.41 | 906 | 22.00 | 22766 | 21.26 |
| - 5 (most deprived) | 86 | 33.08 | 1872 | 29.93 | 907 | 22.03 | 23314 | 21.77 |
| Unknown | 0 | 0.00 | 15 | 0.24 | 16 | 0.39 | 438 | 0.41 |
| Townsend score, mean [SD] | 1.13 [3.46] |  | 1.04 [3.26] |  | 0.24 [3.22] |  | 0.25 [3.16] |  |
| Townsend rank for Wales, mean [SD] | 3.49 [1.40] |  | 3.44 [1.37] |  | 3.10 [1.43] |  | 3.10 [1.41] |  |
| Smoking status |  |  |  |  |  |  |  |  |
| - non-smoker | 102 | 39.23 | 2409 | 38.52 | 2379 | 57.77 | 61047 | 57.01 |
| - current smoker | 125 | 48.08 | 2890 | 46.21 | 1188 | 28.85 | 30642 | 28.61 |
| - ex-smoker | 28 | 10.77 | 886 | 14.17 | 471 | 11.44 | 13193 | 12.32 |
| - unknown | 5 | 1.92 | 69 | 1.10 | 80 | 1.94 | 2203 | 2.06 |

#### Table Ba SSRI* exposures 91 days either side of LMP and outcomes: 3 countries combined

|  | All SSRIs N06AB | | | | fluoxetine N06AB03 | | citalopram N06AB04 | | paroxetine N06AB05 | | sertraline N06AB06 | | escitalopram N06AB10 | |
| --- | --- | --- | --- | --- | --- | --- | --- | --- | --- | --- | --- | --- | --- | --- |
| Outcomes | exposed n=  12,962 | | unexposed n=506,155 | | exposed n=2601 | | exposed n=4028 | | exposed n=1069 | | exposed n=1374 | | exposed n=3236 | |
| EUROCAT subgroup | N | % exposed | n | % unexposed | n | % exposed | N | % exposed | n | % exposed | n | % exposed | n | % exposed |
| al1: All Anomalies | 400 | 3.09 | 13536 | 2.67 | 81 | 3.11 | 136 | 3.38 | 36 | 3.37 | 47 | 3.42 | 86 | 2.66 |
| al3: Neural Tube Defects | 18 | 0.14 | 449 | 0.09 | 7 | 0.27 | 4 | 0.10 | 2 | 0.19 | 0 | 0.00 | 4 | 0.12 |
| al4: Anencephalus and similar | 7 | 0.05 | 170 | 0.03 | 5 | 0.19 | 2 | 0.05 | 0 | 0.00 | 0 | 0.00 | 0 | 0.00 |
| al5: Encephalocele | 4 | 0.03 | 49 | 0.01 | 1 | 0.04 | 1 | 0.02 | 0 | 0.00 | 0 | 0.00 | 2 | 0.06 |
| al6: Spina Bifida | 7 | 0.05 | 230 | 0.05 | 1 | 0.04 | 1 | 0.02 | 2 | 0.19 | 0 | 0.00 | 2 | 0.06 |
| al7: Hydrocephaly | 5 | 0.04 | 245 | 0.05 | 1 | 0.04 | 3 | 0.07 | 0 | 0.00 | 0 | 0.00 | 1 | 0.03 |
| al10: Eye | 14 | 0.11 | 303 | 0.06 | 4 | 0.15 | 5 | 0.12 | 2 | 0.19 | 1 | 0.07 | 1 | 0.03 |
| al11: An-/ micro-ophthalmos | 3 | 0.02 | 46 | 0.01 | 1 | 0.04 | 1 | 0.02 | 0 | 0.00 | 0 | 0.00 | 1 | 0.03 |
| al15: Ear, face and neck | 5 | 0.04 | 116 | 0.02 | 1 | 0.04 | 0 | 0.00 | 0 | 0.00 | 2 | 0.15 | 2 | 0.06 |
| al17: Congenital heart defects | 121 | 0.93 | 4503 | 0.89 | 21 | 0.81 | 40 | 0.99 | 17 | 1.59 | 17 | 1.24 | 21 | 0.65 |
| al97: Severe CHD | 34 | 0.26 | 865 | 0.17 | 8 | 0.31 | 15 | 0.37 | 3 | 0.28 | 5 | 0.36 | 3 | 0.09 |
| al19: Transposition of great vessels (complete) | 3 | 0.02 | 197 | 0.04 | 1 | 0.04 | 0 | 0.00 | 1 | 0.09 | 0 | 0.00 | 1 | 0.03 |
| al21: Ventricular septal defect | 52 | 0.40 | 2218 | 0.44 | 4 | 0.15 | 16 | 0.40 | 12 | 1.12 | 6 | 0.44 | 11 | 0.34 |
| al22: Atrial septal defect | 27 | 0.21 | 1057 | 0.21 | 4 | 0.15 | 5 | 0.12 | 1 | 0.09 | 5 | 0.36 | 10 | 0.31 |
| al24: Tetralogy of Fallot | 7 | s0.05 | 119 | 0.02 | 2 | 0.08 | 2 | 0.05 | 0 | 0.00 | 2 | 0.15 | 1 | 0.03 |
| al27: Pulmonary valve stenosis | 10 | 0.08 | 233 | 0.05 | 6 | 0.23 | 3 | 0.07 | 1 | 0.09 | 0 | 0.00 | 0 | 0.00 |
| al28: Pulmonary valve atresia | 3 | 0.02 | 59 | 0.01 | 0 | 0.00 | 2 | 0.05 | 0 | 0.00 | 1 | 0.07 | 0 | 0.00 |
| al29: Aortic valve atresia/stenosis | 6 | 0.05 | 95 | 0.02 | 1 | 0.04 | 3 | 0.07 | 1 | 0.09 | 0 | 0.00 | 1 | 0.03 |
| al30: Hypoplastic left heart | 6 | 0.05 | 124 | 0.02 | 1 | 0.04 | 3 | 0.07 | 1 | 0.09 | 1 | 0.07 | 0 | 0.00 |
| al32 : Coarctation of aorta | 4 | 0.03 | 158 | 0.03 | 1 | 0.04 | 3 | 0.07 | 0 | 0.00 | 0 | 0.00 | 0 | 0.00 |
| al100: PDA as only CHD in term infants | 8 | 0.06 | 425 | 0.08 | 0 | 0.00 | 2 | 0.05 | 0 | 0.00 | 3 | 0.22 | 2 | 0.06 |
| al101: Oro‐facial clefts | 22 | 0.17 | 861 | 0.17 | 2 | 0.08 | 5 | 0.12 | 1 | 0.09 | 4 | 0.29 | 9 | 0.28 |
| al102 : Cleft lip with or without palate | 15 | 0.12 | 563 | 0.11 | 2 | 0.08 | 3 | 0.07 | 1 | 0.09 | 2 | 0.15 | 6 | 0.19 |
| al103: Cleft palate | 7 | 0.05 | 298 | 0.06 | 0 | 0.00 | 2 | 0.05 | 0 | 0.00 | 2 | 0.15 | 3 | 0.09 |
| al44: Ano‐rectal atresia and stenosis | 7 | 0.05 | 150 | 0.03 | 2 | 0.08 | 1 | 0.02 | 0 | 0.00 | 2 | 0.15 | 1 | 0.03 |
| al45: Hirschsprung's defects | 4 | 0.03 | 74 | 0.01 | 2 | 0.08 | 1 | 0.02 | 0 | 0.00 | 0 | 0.00 | 1 | 0.03 |
| al48: Diaphragmatic hernia | 8 | 0.06 | 128 | 0.03 | 1 | 0.04 | 4 | 0.10 | 0 | 0.00 | 1 | 0.07 | 2 | 0.06 |
| al49: Abdominal wall defects | 15 | 0.12 | 275 | 0.05 | 3 | 0.12 | 3 | 0.07 | 1 | 0.09 | 1 | 0.07 | 6 | 0.19 |
| al50: Gastroschisis | 9 | 0.07 | 161 | 0.03 | 1 | 0.04 | 2 | 0.05 | 0 | 0.00 | 1 | 0.07 | 4 | 0.12 |
| al51: Omphalocele | 3 | 0.02 | 92 | 0.02 | 2 | 0.08 | 0 | 0.00 | 0 | 0.00 | 0 | 0.00 | 1 | 0.03 |
| al52: Urinary | 50 | 0.39 | 1496 | 0.30 | 13 | 0.50 | 19 | 0.47 | 5 | 0.47 | 4 | 0.29 | 7 | 0.22 |
| al54: Renal Dysplasia | 10 | 0.08 | 177 | 0.03 | 3 | 0.12 | 3 | 0.07 | 1 | 0.09 | 0 | 0.00 | 1 | 0.03 |
| al55: Congenital hydronephrosis | 27 | 0.21 | 871 | 0.17 | 6 | 0.23 | 11 | 0.27 | 2 | 0.19 | 4 | 0.29 | 3 | 0.09 |
| al59: Hypospadias | 36 | 0.28 | 1141 | 0.23 | 3 | 0.12 | 17 | 0.42 | 4 | 0.37 | 5 | 0.36 | 7 | 0.22 |
| al62: Limb reduction | 6 | 0.05 | 254 | 0.05 | 2 | 0.08 | 2 | 0.05 | 0 | 0.00 | 1 | 0.07 | 1 | 0.03 |
| al63: Upper limb reduction | 6 | 0.05 | 192 | 0.04 | 2 | 0.08 | 2 | 0.05 | 0 | 0.00 | 1 | 0.07 | 1 | 0.03 |
| al66: talipes equinovarus / clubfoot | 24 | 0.19 | 739 | 0.15 | 3 | 0.12 | 6 | 0.15 | 1 | 0.09 | 3 | 0.22 | 10 | 0.31 |
| al67: Hip dislocation and/or dysplasia | 17 | 0.13 | 754 | 0.15 | 6 | 0.23 | 4 | 0.10 | 1 | 0.09 | 2 | 0.15 | 4 | 0.12 |
| al68: Polydactyly | 10 | 0.08 | 434 | 0.09 | 4 | 0.15 | 3 | 0.07 | 1 | 0.09 | 2 | 0.15 | 0 | 0.00 |
| al69: Syndactyly | 12 | 0.09 | 293 | 0.06 | 2 | 0.08 | 5 | 0.12 | 1 | 0.09 | 1 | 0.07 | 3 | 0.09 |
| al75: Craniosynostosis | 4 | 0.03 | 115 | 0.02 | 0 | 0.00 | 1 | 0.02 | 1 | 0.09 | 1 | 0.07 | 1 | 0.03 |

Notes: Exclusions and exposures* as Table 1.

‘al’ numbers refer to EUROCAT Subgroup binary variable numbers (al). Each number refers to specific ICD9 and ICD10 codes, as listed in the EUROCAT guide 1.3 (EUROCAT 2005)

A fuller version of this table is presented in EMC (2015) [47} in supplementary Tables S3 and S4.

The only other SSRI in the prescription records was fluvoxamine. No infants with congenital anomalies had been exposed to fluvoxamine, and only 28 exposures were identified in the 3 databases, 95% exact binomial confidence intervals for prevalence 0-0.123%.

There were >5 cases of all outcomes. We did not analyse the following outcomes because there were <3 exposed cases: for all SSRI exposure, microcephaly, arhinencephaly, anophthalmos, congenital cataracts, congenital glaucoma, anotia, common arterial truncus, single ventricle, AV septal defect, tricuspid valve atresia/ stenosis, Ebstein’s anomaly, hypoplastic R heart, total anomalous pulmonary venous return, choanal atresia, oesophageal fistula, duodenal or small intestine atresia/ stenosis, atresia of bile ducts, annular pancreas, bilateral renal agenesis, bladder exstrophy, posterior urethral valve, indeterminate sex, lower limb reduction, complete absence of limb, situs inversus, conjoined twins.

We are not reporting heterogeneous groupings: nervous system, al2; respiratory system, al34; digestive system, al40; genital system al58; all limb defects, al61; skin defects al81.

#### Table Bb. Exposure to all antidepressants (ATC N06A) and SNRIs: Wales and Norway. Outcomes with at least 3 exposed cases

|  | All N06A antidepressants | | | | | | SNRI exposed* | | |
| --- | --- | --- | --- | --- | --- | --- | --- | --- | --- |
| Outcomes | Exposed LMP±91 days | N= 15,634 | unexposed  LMP±91 days | N=447,036 | whole population | N=462,670 | Exposed LMP±91 days | N=1448 |  |
| EUROCAT subgroup | N | % exposed | n | % unexposed | n | % population | n | % exposed | |
| al1: All Anomalies | 462 | 2.96 | 12,186 | 2.73 | 12,648 | 2.73 | 46 | 3.18 |  |
| al3: Neural Tube Defects | 20 | 0.13 | 390 | 0.09 | 410 | 0.09 | 0 | 0.00 |  |
| al4: Anencephalus and similar | 7 | 0.04 | 146 | 0.03 | 153 | 0.03 | 0 | 0.00 |  |
| al5: Encephalocele | 5 | 0.03 | 42 | 0.01 | 47 | 0.01 | 0 | 0.00 |  |
| al6: Spina Bifida | 8 | 0.05 | 202 | 0.05 | 210 | 0.05 | 0 | 0.00 |  |
| al7: Hydrocephaly | 8 | 0.05 | 218 | 0.05 | 226 | 0.05 | 0 | 0.00 |  |
| al8: Microcephaly | 3 | 0.02 | 62 | 0.01 | 65 | 0.01 | 1 | 0.07 |  |
| al10: Eye | 16 | 0.10 | 268 | 0.06 | 284 | 0.06 | 0 | 0.00 |  |
| al11: Anophthalmos/microphthalmos | 3 | 0.02 | 32 | 0.01 | 35 | 0.01 | 0 | 0.00 |  |
| al15: Ear, face and neck | 5 | 0.03 | 112 | 0.03 | 117 | 0.03 | 0 | 0.00 |  |
| al17: Congenital heart defects | 136 | 0.87 | 4025 | 0.90 | 4161 | 0.90 | 18 | 1.24 |  |
| al97: Severe CHD | 31 | 0.20 | 764 | 0.17 | 795 | 0.17 | 1 | 0.07 |  |
| al21: Ventricular septal defect | 57 | 0.36 | 1939 | 0.43 | 1996 | 0.43 | 8 | 0.55 |  |
| al22: Atrial septal defect | 29 | 0.19 | 998 | 0.22 | 1027 | 0.22 | 1 | 0.07 |  |
| al24: Tetralogy of Fallot | 7 | 0.04 | 101 | 0.02 | 108 | 0.02 | 0 | 0.00 |  |
| al27: Pulmonary valve stenosis | 13 | 0.08 | 193 | 0.04 | 206 | 0.04 | 3 | 0.21 |  |
| al28: Pulmonary valve atresia | 3 | 0.02 | 56 | 0.01 | 59 | 0.01 | 0 | 0.00 |  |
| al29: Aortic valve atresia/stenosis | 7 | 0.04 | 84 | 0.02 | 91 | 0.02 | 1 | 0.07 |  |
| al30: Hypoplastic left heart | 5 | 0.03 | 108 | 0.02 | 113 | 0.02 | 0 | 0.00 |  |
| al32: Coarctation of aorta | 3 | 0.02 | 143 | 0.03 | 146 | 0.03 | 0 | 0.00 |  |
| al100: PDA as only CHD in term infants | 13 | 0.08 | 409 | 0.09 | 422 | 0.09 | 3 | 0.21 |  |
| al101: Oro‐facial clefts | 24 | 0.15 | 759 | 0.17 | 783 | 0.17 | 3 | 0.21 |  |
| al102: Cleft lip with or without palate | 15 | 0.10 | 495 | 0.11 | 510 | 0.11 | 1 | 0.07 |  |
| al103: Cleft palate | 9 | 0.06 | 264 | 0.06 | 273 | 0.06 | 2 | 0.14 |  |
| al44: Ano‐rectal atresia and stenosis | 7 | 0.04 | 129 | 0.03 | 136 | 0.03 | 0 | 0.00 |  |
| al45: Hirschsprung's disease | 5 | 0.03 | 62 | 0.01 | 67 | 0.01 | 1 | 0.07 |  |
| al48: Diaphragmatic hernia | 8 | 0.05 | 118 | 0.03 | 126 | 0.03 | 0 | 0.00 |  |
| al49 Abdominal wall defects | 17 | 0.11 | 251 | 0.06 | 268 | 0.06 | 1 | 0.07 |  |
| al50: Gastroschisis | 11 | 0.07 | 152 | 0.03 | 163 | 0.04 | 1 | 0.07 |  |
| al51: Omphalocele | 3 | 0.02 | 78 | 0.02 | 81 | 0.02 | 0 | 0.00 |  |
| al53: Bilateral renal agenesis including Potter syndrome | 3 | 0.02 | 38 | 0.01 | 41 | 0.01 | 2 | 0.14 |  |
| al54: Renal Dysplasia | 12 | 0.08 | 142 | 0.03 | 154 | 0.03 | 2 | 0.14 |  |
| al55: Congenital hydronephrosis | 29 | 0.19 | 806 | 0.18 | 835 | 0.18 | 3 | 0.21 |  |
| al59: Hypospadias | 39 | 0.25 | 1015 | 0.23 | 1054 | 0.23 | 2 | 0.14 |  |
| al62: Limb reduction | 8 | 0.05 | 211 | 0.05 | 219 | 0.05 | 2 | 0.14 |  |
| al63: Upper limb reduction | 8 | 0.05 | 161 | 0.04 | 169 | 0.04 | 2 | 0.14 |  |
| al66: Talipes equinovarus / Clubfoot | 30 | 0.19 | 656 | 0.15 | 686 | 0.15 | 3 | 0.21 |  |
| al67: Hip dislocation and/or dysplasia | 19 | 0.12 | 665 | 0.15 | 684 | 0.15 | 1 | 0.07 |  |
| al68: Polydactyly | 12 | 0.08 | 399 | 0.09 | 411 | 0.09 | 1 | 0.07 |  |
| al69: Syndactyly | 13 | 0.08 | 270 | 0.06 | 283 | 0.06 | 0 | 0.00 |  |
| al75: Craniosynostosis | 6 | 0.04 | 93 | 0.02 | 99 | 0.02 | 2 | 0.14 |  |
|  |  |  |  |  |  |  |  |  |  |

Notes: *SNRIs identified in the database were: venlafaxine N06AX16, reboxetine N06AX18, duloxetine N06AX21

Exclusions as Table 1

There were >5 cases of all outcomes. We did not analyse the following outcomes because there were <3 exposed cases: for all antidepressant exposure, arhinencephaly, anophthalmos, congenital cataracts, congenital glaucoma, anotia, common arterial truncus, transposition of great vessels, single ventricle, AV septal defect, tricuspid valve atresia/ stenosis, Ebstein’s anomaly, hypoplastic R heart, total anomalous pulmonary venous return, choanal atresia, oesophageal fistula, duodenal or small intestine atresia/ stenosis, atresia of bile ducts, annular pancreas, bladder exstrophy, posterior urethral valve, indeterminate sex, lower limb reduction, complete absence of limb, situs inversus, conjoined twins.

We are not reporting heterogeneous groupings: nervous system, al3; respiratory system, al34; digestive system, al40; genital system al58; all limb defects, al61; skin defects al81.

#### Table C. Congenital anomalies* and antidepressant exposures 91 days either side of LMP: meta-analyses + 3 countries

|  |  | Meta-analysis |  | Denmark | Norway | Wales |  |
| --- | --- | --- | --- | --- | --- | --- | --- |
| EUROCAT subgroup: CA | Exposure | OR (95% CI) | Exposed Cases | OR (95% CI) | OR (95% CI) | OR (95% CI) | I^2^ |
| al1: All Anomalies | All antidepressants | 1.03 (0.93 - 1.13) | 462 |  | 1.00 (0.87 - 1.16) | 1.05 (0.92 - 1.19) | 0.00 |
|  | All SSRIs* | 1.09 (0.99 - 1.21) | 400 | 1.25 (0.88 - 1.77) | 1.06 (0.9 - 1.24) | 1.10 (0.96 - 1.26) | 0.00 |
|  | Fluoxetine | 1.05 (0.84 - 1.31) | 81 | 2.34 (1.16 - 4.72) | 0.45 (0.2 - .98) | 1.11 (0.87 - 1.41) | 78.61 |
|  | Citalopram | 1.16 (0.98 - 1.38) | 136 | 1.20 (0.70 - 2.08) | 1.3 (0.9 - 1.88) | 1.12 (0.91 - 1.38) | 0.00 |
|  | Paroxetine | 1.17 (0.84 - 1.64) | 36 | 0.83 (0.01 - 3.05) | 1.32 (0.73 - 2.38) | 1.15 (0.76 - 1.75) | 0.00 |
|  | Sertraline | 1.27 (0.95 - 1.70) | 47 | 1.01 (0.39 - 2.62) | 1.46 (1.01 - 2.1) | 1.05 (0.61 - 1.82) | 0.00 |
|  | Escitalopram | 1.01 (0.81 - 1.25) | 86 | 1.62 (0.68 - 3.85) | 1.00 (0.79 - 1.26) | 0.91 (0.49 - 1.7) | 0.00 |
|  |  |  |  |  |  |  |  |
| al3: Neural Tube Defects | All antidepressants | 1.33 (0.85 - 2.09) | 20 |  | 1.5 (0.78 - 2.87) | 1.21 (0.66 - 2.23) | 0.00 |
|  | All SSRIs | 1.43 (0.89 - 2.30) | 18 |  |  |  | 0.00 |
|  | Fluoxetine | 2.57 (1.21 - 5.46) | 7 |  |  |  | 0.00 |
|  | Citalopram | 0.96 (0.36 - 2.57) | 4 |  |  |  | 0.00 |
|  | Escitalopram | 1.48 (0.55 - 3.95) | 4 |  |  |  | 0.00 |
|  |  |  |  |  |  |  |  |
| al17: Congenital heart defects | All antidepressants | 0.95 (0.8 - 1.13) | 136 |  | 0.93 (0.73 - 1.2) | 0.97 (0.76 - 1.23) | 0.00 |
|  | All SSRIs | 1.03 (0.86 - 1.24) | 121 | 1.70 (1.04 - 2.8) | 0.91 (0.68 - 1.22) | 1.02 (0.79 - 1.33) | 55.66 |
|  | Fluoxetine | 0.87 (0.57 - 1.35) | 21 | 1.60 (0.01 - 5.91) | NA (NA - 0.84) | 1.05 (0.66 - 1.64) | 0.00 |
|  | Citalopram | 1.09 (0.8 - 1.49) | 40 | 1.82 (0.87 - 3.80) | 1.30 (0.71 - 2.41) | 0.91 (0.60 - 1.38) | 28.99 |
|  | Paroxetine | 1.76 (1.09 - 2.85) | 17 |  |  |  | 0.00 |
|  | Sertraline | 1.39 (0.86 - 2.25) | 17 |  |  |  | 0.00 |
|  | Escitalopram | 0.73 (0.47 - 1.12) | 21 |  |  |  | 65.96 |
|  |  |  |  |  |  |  |  |
| al97: Severe CHD | All antidepressants | 1.12 (0.78 - 1.61) | 31 |  | 0.79 (0.42 - 1.46) | 1.43 (0.91 - 2.23) | 57.43 |
|  | All SSRIs | 1.50 (1.06 - 2.11) | 34 | 2.90 (1.3 - 6.49) | 0.99 (0.52 - 1.90) | 1.64 (1.03 - 2.62) | 51.36 |
|  | Fluoxetine | 1.72 (0.85 - 3.47) | 8 |  |  |  | 0.00 |
|  | Citalopram | 2.09 (1.25 - 3.51) | 15 |  |  |  | 0.00 |
|  | Paroxetine | 1.59 (0.51 - 4.95) | 3 |  |  |  | 0.00 |
|  | Sertraline | 2.12 (0.88 - 5.11) | 5 |  |  |  | 0.00 |
|  | Escitalopram | 0.55 (0.18 - 1.71) | 3 |  |  |  | 51.51 |
|  |  |  |  |  |  |  |  |
| al21: Ventricular septal defect | All antidepressants | 0.85 (0.65 - 1.10) | 57 |  | 0.87 (0.60 - 1.26) | 0.82 (0.56 - 1.20) | 0.00 |
|  | All SSRIs | 0.93 (0.7 - 1.22) | 52 | 1.80 (0.96 - 3.36) | 0.88 (0.58 - 1.35) | 0.78 (0.50 - 1.20) | 58.73 |
|  | Fluoxetine | 0.36 (0.13 - 0.95) | 4 |  |  |  | 0.00 |
|  | Citalopram | 0.92 (0.56 - 1.51) | 16 |  |  |  | 0.00 |
|  | Paroxetine | 2.61 (1.47 - 4.62) | 12 |  |  |  | 0.00 |
|  | Sertraline | 1.00 (0.45 - 2.23) | 6 |  |  |  | 0.00 |
|  | Escitalopram | 0.78 (0.43 - 1.41) | 11 |  |  |  | 79.07 |
|  |  |  |  |  |  |  |  |
| al27: Pulmonary valve stenosis | All antidepressants | 1.23 (0.70 - 2.17) | 13 |  |  |  | 0.00 |
|  | All SSRIs | 1.10 (0.58 - 2.09) | 10 |  |  |  | 0.00 |
|  | Fluoxetine | 2.52 (1.11 - 5.7) | 6 |  |  |  | 0.00 |
|  | Citalopram | 0.86 (0.28 - 2.69) | 3 |  |  |  | 65.81 |
|  |  |  |  |  |  |  |  |
| al44: Ano-rectal atresia and stenosis | All antidepressants | 1.56 (0.73 - 3.36) | 7 |  |  |  | 0.00 |
|  | All SSRIs | 1.85 (0.86 - 3.96) | 7 |  |  |  | 0.00 |
|  |  |  |  |  |  |  |  |
| al49: Abdominal wall defects | All antidepressants | 1.75 (1.07 - 2.88) | 17 |  | 2.07 (1.03 - 4.15) | 1.53 (0.78 - 3.02) | 0.00 |
|  | All SSRIs | 1.92 (1.13 - 3.24) | 15 |  |  |  | 0.00 |
|  | Fluoxetine | 1.74 (0.55 - 5.46) | 3 |  |  |  | 60.51 |
|  | Citalopram | 1.16 (0.37 - 3.64) | 3 |  |  |  | 0.00 |
|  | Escitalopram | 3.52 (1.56 - 7.91) | 6 |  |  |  | 0.00 |
|  |  |  |  |  |  |  |  |
| Al50: Gastroschisis | All antidepressants | 1.85 (1.00 - 3.42) | 11 |  |  |  | 0.00 |
|  | All SSRIs | 1.92 (0.97 - 3.78) | 9 |  |  |  | 12.64 |
|  | Escitalopram | 3.95 (1.46 - 10.67) | 4 |  |  |  | 12.64 |
|  |  |  |  |  |  |  |  |
| al54: Renal Dysplasia | All antidepressants | 1.65 (0.91 - 2.99) | 12 |  |  |  | 0.00 |
|  | All SSRIs | 1.57 (0.83 - 2.98) | 10 |  |  |  | 0.00 |
|  | Fluoxetine | 1.87 (0.60 - 5.86) | 3 |  |  |  | 83.91 |
|  | Citalopram | 1.23 (0.39 - 3.89) | 3 |  |  |  | 0.00 |
|  |  |  |  |  |  |  |  |
| al59: Hypospadias | All antidepressants | 1.03 (0.74 - 1.42) | 39 |  | 0.80 (0.46 - 1.37) | 1.21 (0.81 - 1.8) | 30.56 |
|  | All SSRIs | 1.15 (0.82 - 1.61) | 36 | 1.18 (0.40 - 3.52) | 1.03 (0.59 - 1.82) | 1.23 (0.79 - 1.91) | 0.00 |
|  | Fluoxetine | 0.45 (0.14 - 1.4) | 3 |  |  |  | 0.00 |
|  | Citalopram | 1.69 (1.04 - 2.73) | 17 | 1.93 (0.01 - 7.13) | 1.63 (0.55 - 4.81) | 1.66 (0.94 - 2.95) | 0.00 |
|  | Paroxetine | 1.52 (0.57 - 4.06) | 4 |  |  |  | 0.00 |
|  | Sertraline | 1.59 (0.66 - 3.83) | 5 |  |  |  | 0.00 |
|  | Escitalopram | 0.99 (0.47 - 2.08) | 7 |  |  |  | 0.00 |
|  |  |  |  |  |  |  |  |
| al62: Limb reduction | All antidepressants | 0.93 (0.46 - 1.89) | 8 |  |  |  | 59.57 |
|  | All SSRIs | 0.81 (0.36 - 1.82) | 6 |  |  |  | 0.00 |
|  |  |  |  |  |  |  |  |
| al66: talipes equinovarus / Clubfoot | All antidepressants | 1.23 (0.85 - 1.78) | 30 |  | 1.62 (1.00 - 2.61) | 0.93 (0.53 - 1.62) | 53.83 |
|  | All SSRIs | 1.20 (0.79 - 1.80) | 24 | 0.62 (0.01 - 3.56) | 1.59 (0.90 - 2.8) | 1.00 (0.55 - 1.82) | 0.00 |
|  | Fluoxetine | 0.70 (0.23 - 2.19) | 3 |  |  |  | 45.33 |
|  | Citalopram | 0.92 (0.41 - 2.06) | 6 |  |  |  | 0.00 |
|  | Sertraline | 1.47 (0.47 - 4.58) | 3 |  |  |  | 0.00 |
|  | Escitalopram | 2.18 (1.16 - 4.07) | 10 |  |  |  | 0.00 |
|  |  |  |  |  |  |  |  |
| Al101 Oro-facial clefts | All antidepressants | 0.95 (0.63 - 1.43) | 24 |  | 0.89 (0.51 - 1.56) | 1.02 (0.57 - 1.81) | 0.00 |
|  | All SSRIs | 1.05 (0.68 - 1.60) | 22 | 0.96 (0.01 - 3.56) | 0.93 (0.49 - 1.79) | 1.19 (0.65 - 2.17) | 0.00 |
|  | Citalopram | 0.79 (0.33 - 1.91) | 5 |  |  |  | 0.00 |
|  | Sertraline | 1.74 (0.65 - 4.64) | 4 |  |  |  | 0.00 |
|  | Escitalopram | 1.61 (0.83 - 3.1) | 9 |  |  |  | 0.00 |

Table notes: Exclusions as Table 1

Notes: Exclusions and *exposure as in Table 1.

Results are not shown where there were <3 exposed cases across the 3 countries, and no meta-analyses were undertaken. Data were redacted where 1-4 exposed cases occurred in any country, to comply with data disclosure regulations.

No associations were statistically significant in all 3 countries.

Denmark did not supply all exposure data, reducing the numbers in the analysis and the numbers of exposed cases in the ‘all antidepressant’ (N06A) category.

Further information is in Tables Ba, Bb (including numbers and %s of cases), and EMC 2015 [41] supplementary tables S3 and S4.

Data on SNRIs from Wales and Norway are presented in EMC (2015) [41], and Table Bb. (Denmark was unable to supply data on SNRIs.) There were 1448 SNRI exposures and 46 exposed cases (3.18%) (OR 1.14, 0.85 - 1.53). No statistically significant associations with the anomalies listed above were identified.

An analysis of SNRI exposure in Wales and Norway can be found in EMC (2015) [41]

Rubrics indicate 95% confidence intervals exclude 1.

#### Table D. SSRI exposure 91 days either side of LMP with and without other antidepressants: outcomes with at least 4 exposed cases

|  | SSRI exposed, all, 3 countries | | | | | | meta-analysis |  | SSRI exposed, those exposed to other antidepressants excluded,  Norway & Wales only | | | | | | meta-analysis |  |
| --- | --- | --- | --- | --- | --- | --- | --- | --- | --- | --- | --- | --- | --- | --- | --- | --- |
| outcome | Exposed n=12,962 | | Unexposed n=506,115 | | Population n=519,117 | | OR (95% CI) | I^2^ | Exposed n=10,959 | | Unexposed n=449,643 | | Population n=460,602 | | OR (95% CI) | I^2^ |
| EUROCAT subgroup | n | % | n | % | n | % |  |  | n | % | n | % | n | % |  |  |
| al1: All Anomalies | 400 | 3.09 | 13,536 | 2.67 | 13,936 | 2.68 | 1.09 (0.99 - 1.21) | 0 | 341 | 3.11 | 12,247 | 2.72 | 12,588 | 2.73 | 1.08 (0.97 - 1.20) | 0 |
| al3: Neural Tube Defects | 18 | 0.14 | 449 | 0.09 | 467 | 0.09 | 1.43 (0.89 - 2.30) | 0 | 13 | 0.12 | 395 | 0.09 | 408 | 0.09 | 1.21 (0.69 - 2.11) | 0 |
| al4: Anencephalus and similar | 7 | 0.05 | 170 | 0.03 | 177 | 0.03 | 1.35 (0.63 - 2.90) | 14.55 | 4 | 0.04 | 148 | 0.03 | 152 | 0.03 | 0.90 (0.33 - 2.45) |  |
| al10: Eye | 14 | 0.11 | 303 | 0.06 | 317 | 0.06 | 1.07 (0.62 - 1.83) | 0 | 12 | 0.11 | 269 | 0.06 | 281 | 0.06 | 1.02 (0.57 - 1.83) | 0 |
| al17: Congenital heart defects | 121 | 0.93 | 4503 | 0.89 | 4624 | 0.89 | 1.03 (0.86 - 1.24) | 55.66 | 101 | 0.92 | 4047 | 0.9 | 4148 | 0.9 | 1.00 (0.82 - 1.22) | 0 |
| al97: Severe CHD | 34 | 0.26 | 865 | 0.17 | 899 | 0.17 | 1.5 (1.06 - 2.11) | 51.36 | 26 | 0.24 | 766 | 0.17 | 792 | 0.17 | 1.34 (0.91 – 2.00) | 37.32 |
| al21: Ventricular septal defect | 52 | 0.4 | 2218 | 0.44 | 2270 | 0.44 | 0.93 (0.7 - 1.22) | 58.73 | 41 | 0.37 | 1948 | 0.43 | 1989 | 0.43 | 0.87 (0.64 - 1.19) | 0 |
| al22 Atrial septal defect | 27 | 0.21 | 1057 | 0.21 | 1084 | 0.21 | 1.01 (0.69 - 1.48) | 56.68 | 23 | 0.21 | 1003 | 0.22 | 1026 | 0.22 | 0.96 (0.63 - 1.45) | 41.84 |
| al24: Tetralogy of Fallot | 7 | 0.05 | 119 | 0.02 | 126 | 0.02 | 2.13 (0.99 - 4.57) | 0 | 5 | 0.05 | 102 | 0.02 | 107 | 0.02 | 1.82 (0.74 - 4.47) | 71.44 |
| al27: Pulmonary valve stenosis | 10 | 0.08 | 233 | 0.05 | 243 | 0.05 | 1.10 (0.58 - 2.09) | 0 | 9 | 0.08 | 193 | 0.04 | 202 | 0.04 | 1.18 (0.6 - 2.3) | 0 |
| al30: Hypoplastic left heart | 6 | 0.05 | 124 | 0.02 | 130 | 0.03 | 2.13 (0.93 - 4.92) | 26.30 | 4 | 0.04 | 109 | 0.02 | 113 | 0.02 | 1.71 (0.62 - 4.74) | 63.51 |
| al44: Ano-rectal atresia and stenosis | 7 | 0.05 | 150 | 0.03 | 162 | 0.03 | 1.56 (0.73- 3.36) | 0 | 4 | 0.04 | 132 | 0.03 | 136 | 0.03 | 1.24 (.45 - 3.4) | 0 |
| al48: Diaphragmatic hernia | 8 | 0.06 | 128 | 0.03 | 136 | 0.03 | 2.31 (1.12 - 4.75) | 0 | 8 | 0.07 | 118 | 0.03 | 126 | 0.03 | 2.62 (1.27 - 5.41) | 0 |
| al49: Abdominal wall defects | 15 | 0.12 | 275 | 0.05 | 290 | 0.06 | 1.92 (1.13 - 3.24) | 0 | 14 | 0.13 | 253 | 0.06 | 267 | 0.06 | 2.03 (1.18 - 3.5) | 0 |
| al50: Gastroschisis | 9 | 0.07 | 161 | 0.03 | 170 | 0.03 | 1.92 (0.97 - 3.78) | 0 | 9 | 0.08 | 154 | 0.03 | 163 | 0.04 | 2.11 (1.07 - 4.16) | 0 |
| al54: Renal Dysplasia | 10 | 0.08 | 177 | 0.03 | 187 | 0.04 | 1.57 (0.83 - 2.98) | 0 | 10 | 0.09 | 142 | 0.03 | 152 | 0.03 | 1.9 (1 - 3.63) | 0 |
| al59: Hypospadias | 36 | 0.28 | 1141 | 0.23 | 1177 | 0.23 | 1.15 (0.82 - 1.61) | 0 | 30 | 0.27 | 1017 | 0.23 | 1047 | 0.23 | 1.12 (0.78 - 1.62) | 0 |
| al62: Limb reduction | 6 | 0.05 | 254 | 0.05 | 260 | 0.05 | 0.81 (0.36 - 1.82) | 0 | 6 | 0.05 | 213 | 0.05 | 219 | 0.05 | 0.97 (0.43 - 2.2) | 0 |
| al66: Club foot ‐ talipes equinovarus | 24 | 0.19 | 739 | 0.15 | 763 | 0.15 | 1.20 (0.79 - 1.8) | 0 | 21 | 0.19 | 661 | 0.15 | 682 | 0.15 | 1.22 (0.79 - 1.89) | 58.47 |
| al69: Syndactyly | 12 | 0.09 | 293 | 0.06 | 305 | 0.06 | 1.67 (0.93 - 2.98) | 0 | 12 | 0.11 | 271 | 0.06 | 283 | 0.06 | 1.91 (1.06 - 3.41) | 0 |
| al101: Oro‐facial clefts | 22 | 0.17 | 861 | 0.17 | 883 | 0.17 | 1.05 (0.68 - 1.60) | 0 | 18 | 0.16 | 763 | 0.17 | 781 | 0.17 | 1.02 (0.64 - 1.63) | 0 |

Notes: Exclusions as Table 1.

We are not reporting heterogeneous groupings: nervous system, al2; respiratory system, al34; digestive system, al40; genital system al58; all limb defects, al61; skin defects al81.

The 2 sets of findings are juxtaposed for convenience.

#### Table E. Deprivation and selected exposures in Wales

| Townsend fifth | Population n (%) | Depression diagnosed ever n (% exposed) | Any SSRI prescription in pre1 or tri1 n (% exposed) | >1 SSRI prescription in pre1 or tri1 n (% exposed) |
| --- | --- | --- | --- | --- |
| 1 least deprived | 18,073 (17.5) | 1740 (9.6) | 684(3.8) | 439 (2.4) |
| 2 | 19,734 (19.1) | 2040 (10.3) | 848 (4.3) | 529 (2.7) |
| 3 | 20,147 (19.5) | 2447 (12.1) | 1069 (5.3) | 641 (3.2) |
| 4 | 22,198 (21.5) | 3160 (14.2) | 1322 (6.0) | 789 (3.6) |
| 5 most deprived | 22,661 (22.0) | 3777 (16.7) | 1670 (7.4) | 1054 (4.7) |
| Unknown | 391 (0.4) | 25 (6.4) | 12 (3.1) | 4 (1.0) |
| Total | 103,204 | 13,189 (12.8) | 5605 (5.4) | 3456 (3.3) |
| χ^2^ df=1 (unknown removed) |  | 603.59 | 313.84 | 182.93 |
| P value |  | <0.001 | <0.001 | <0.001 |

Notes: Exclusions as Table 7

Deprivation (Townsend) scores, ranks and fifths are based on geographical area of residence, using Lower Super Output Areas (LSOAs) defined by postcodes. This measure of material deprivation is calculated from rates of unemployment, vehicle ownership, home ownership, and overcrowding.

#### Table F. Exploration of anomalies and alternative exposures in Wales

|  | Population n (%) | All anomalies  n (% of cases) | OR (95% CI) | Anomaly or stillbirth n (% of cases) | OR (95% CI) | Abdominal wall defects n (% of cases) | OR (95% CI) | CHD  n (% of cases) | OR (95% CI) | Severe CHD n (% of cases) | OR (95% CI) |
| --- | --- | --- | --- | --- | --- | --- | --- | --- | --- | --- | --- |
| Smoking status closest to LMP (ex-smokers removed) | | | |  |  |  |  |  |  |  |  |
| Smoking | 30,534 (34.3) | 996 (34.8) | 1.02 (0.95-1.11) | 1129 (35.4) | 1.05 (0.98-1.13) | 34 (52.3) | 2.10 (1.29-3.42) | 288 (33.9) | 0.98 (0.85-1.13) | 56 (34.8) | 1.02 (0.74-1.41) |
| Not smoking | 58,497 (65.7) | 1866 (65.2) |  | 2061 (64.6) |  | 31 (47.7) |  | 501 (66.1) |  | 105 (65.2) |  |
|  |  |  |  |  |  |  |  |  |  |  |  |
| Heavy drinking and/or substance misuse | | | |  |  |  |  |  |  |  |  |
| Recorded | 1658 (1.6) | 56 (1.7) | 1.07 (0.82-1.40) | 63 (1.7) | 1.08 (0.84-1.39) | 5 (6.6) | 4.32 (1.74-10.72) | 19 (1.9) | 1.22 (0.77-1.92) | <5 | <1 (P>0.05) |
| Not recorded | 101546 (98.4) | 3210 (98.3) |  | 3581 (98.3) |  | 71 (93.4) |  | 959 (98.1) |  |  |  |
|  |  |  |  |  |  |  |  |  |  |  |  |
| Any antipsychotic during LMP ± 91 days | | |  |  |  |  |  |  |  |  |  |
| Prescribed | 175 (0.17) | 8 (0.2) | 1.47 (0.72-2.98) | 8 (0.2) | 1.31 (0.64-2.65) | 0 |  | <5 | >1 (P>0.05) | <5 | >1 (P>0.05) |
| Not prescribed | 103029 (99.83) | 3258 (99.8) |  | 3636 (99.8) |  |  |  |  |  |  |  |
|  |  |  |  |  |  |  |  |  |  |  |  |
| Townsend fifth | Population n (%) | Al1 All anomalies  n (% of cases) | χ^2^ df=1 (unknown removed) (P value) | Anomaly or stillbirth | χ^2^ df=1 (unknown removed) (P value) | Al49 Abdominal wall defects n (% of cases) | χ^2^ df=1 (unknown removed) (P value) | CHD  n (% of cases) | χ^2^ df=1 (unknown removed) (P value) | Severe CHD n (% of cases) | χ^2^ df=1 (unknown removed) (P value) |
| 1 least deprived | 18073 (17.5) | 621 (19.0) | 1.01 (0.32) | 681 (18.7) | 2.20 (0.14) | 9 (11.8) | 11.95 (0.001) | 179 (18.3) | 0.87 (0.35) | 31 (15.8) | 0.58 (0.45) |
| 2 | 19734 (19.1) | 548 (16.8) |  | 612 (16.8) |  | 5 (6.6) |  | 167 (17.1) |  | 36 (18.4) |  |
| 3 | 20147 (19.5) | 606 (18.6) |  | 676 (18.6) |  | 15 (19.7) |  | 181 (18.5) |  | 41 (20.9) |  |
| 4 | 22198 (21.5) | 726 (22.2) |  | 823 (22.6) |  | 20 (26.3) |  | 218 (22.3) |  | 39 (19.9) |  |
| 5 most deprived | 22661 (22.0) | 756 (23.1) |  | 843 (23.2) |  | 27 (35.5) |  | 229 (23.4) |  | 48 (24.5) |  |
| Unknown | 391 (0.4) | 9 (0.3) |  | 9 (0.2) |  | 0 |  | 4 (0.4) |  | 1 (0.5) |  |
| Total | 103204 (100) | 3266 (100) |  | 3644 (100) |  | 76 (100) |  | 978 (100) |  | 196 (100) |  |

Notes: Exclusions as Table 7

There are too few cases of gastroschisis to report as a separate category. Findings are similar to those for all abdominal defects, reported above.

Benzodiazepines: 23 exposures during LMP ± 91 days were identified, 0 exposed cases.

#### Table G. Comparisons of stopping before pregnancy, pausing during pregnancy, exposure LMP±91 days, and unexposed for 11 quarters for all anomalies, CHD and severe CHD, including receipt of >0 and >1 prescriptions: 3 countries

| Exposed >0 SSRI prescription n=11,512 | | | | |  |  | Exposed >1 SSRI prescription n=6392 | | | | |  |  |
| --- | --- | --- | --- | --- | --- | --- | --- | --- | --- | --- | --- | --- | --- |
|  | Stoppers |  | Pausers |  | meta OR (95%CI) | I^2^ |  | Stoppers |  | pausers |  | meta OR (95%CI) | I^2^ |
|  | n | % | n | % |  |  |  | n | % | n | % |  |  |
| Total | 6315 |  | 2203 |  |  |  |  | 3146 |  | 923 |  |  |  |
| all anomalies | 175 | 2.77 | 62 | 2.81 | 0.94 (0.7 - 1.27) | 0 | all anomalies | 87 | 2.77 | 26 | 2.82 | 0.93 (0.59 - 1.46) | 0 |
| CHD | 64 | 1.01 | 24 | 1.09 | 1.04 (0.64 - 1.67) | 0 | CHD | 29 | 0.92 | 11 | 1.19 | 1.33 (0.65 - 2.72) | 0 |
| severe CHD | 9 | 0.14 | 4 | 0.18 | 1.36 (0.4 - 4.66) | 0 | severe CHD | 6 | 0.19 | 3 | 0.33 | 2.37 (0.54 - 10.34) | 37.06 |
|  |  |  |  |  |  |  |  |  |  |  |  |  |  |
|  | Stoppers |  | Exposed LMP±91 days | | meta OR (95%CI) | I^2^ |  | stoppers |  | Exposed LMP±91 days | | meta OR (95%CI) | I^2^ |
|  | n | % | N | % |  |  |  | n | % | n | % |  |  |
| Total | 6315 |  | 11,512 |  |  |  |  | 3146 |  | 6392 |  |  |  |
| all anomalies | 175 | 2.77 | 341 | 2.96 | 0.95 (0.79 - 1.15) | 0 | all anomalies | 87 | 2.77 | 190 | 2.97 | 0.97 (0.75 - 1.26) | 0 |
| CHD | 64 | 1.01 | 94 | 0.82 | 1.27 (0.92 - 1.75) | 55.47 | CHD | 29 | 0.92 | 56 | 0.88 | 1.11 (0.7 - 1.76) | 34.37 |
| severe CHD | 9 | 0.14 | 26 | 0.23 | 0.65 (0.3 - 1.41) | 66.30 | severe CHD | 6 | 0.19 | 20 | 0.31 | 0.60 (0.23 - 1.58) | 66.28 |
|  |  |  |  |  |  |  |  |  |  |  |  |  |  |
|  | pausers |  | exposed LMP±91 days | | meta OR (95%CI) | I^2^ |  | pausers |  | Exposed LMP±91 days | | meta OR (95%CI) | I^2^ |
|  | n | % | n | % |  |  |  | n | % | n | % |  |  |
| Total | 2203 |  | 11,512 |  |  |  | n | 923 |  | 6392 |  |  |  |
| all anomalies | 62 | 2.81 | 341 | 2.96 | 0.91 (0.69 - 1.20) | 0 | all anomalies | 26 | 2.82 | 190 | 2.97 | 0.94 (0.62 - 1.42) | 0 |
| CHD | 24 | 1.09 | 94 | 0.82 | 1.31 (0.83 - 2.07) | 26.38 | CHD | 11 | 1.19 | 56 | 0.88 | 1.35 (0.71 - 2.6) | 0 |
| severe CHD | 4 | 0.18 | 26 | 0.23 | 0.73 (0.25 - 2.10) | 0 | severe CHD | 3 | 0.33 | 20 | 0.31 | 1.00 (0.29 - 3.43) | 0 |
|  |  |  |  |  |  |  |  |  |  |  |  |  |  |
|  | exposed >0 LMP±91 days | | unexposed 11 quarters* | | meta OR (95%CI) | I^2^ |  | exposed >1 LMP±91 days | | unexposed 11 quarters* | | meta OR (95%CI) | I^2^ |
|  | n | % | n | % |  |  |  | n | % | n | % |  |  |
| Total | 11,512 |  | 426,962 |  |  |  |  | 6392 |  | 426,962 |  |  |  |
| all anomalies | 341 | 2.96 | 11,049 | 2.59 | 1.08 (0.97 - 1.20) | 0 | all anomalies | 190 | 2.97 | 11,049 | 2.59 | 1.20 (1.04 - 1.39) | 34.33 |
| CHD | 94 | 0.82 | 3651 | 0.82 | 0.94 (0.76 - 1.15) | 76.63 | CHD | 56 | 0.88 | 3651 | 0.82 | 1.11 (0.85 - 1.45) | 70.35 |
| severe CHD | 26 | 0.23 | 722 | 0.16 | 1.29 (0.87 - 1.92) | 77.72 | severe CHD | 20 | 0.31 | 722 | 0.16 | 1.98 (1.26 - 3.10) | 60.51 |

Notes: Exclusions as Table 1 plus ‘not on database for 1 year either side of pregnancy’. *11 quarters - pregnancy and 1 year either side.

#### Table H. Depression, medicated and unmedicated and congenital anomalies and stillbirths in Wales

|  | depression diagnosed (ever)  n= 13189 | | no depression  recorded  n=90015 | |  | Depression exposed to N06A LMP±91 days  n= 3598 | | Depression un-medicated with N06A LMP±91 days n= 9591 | |  | Depression exposed to N06AB LMP±91 days  n= 2897 | | Depression un-medicated with N06AB* LMP±91 days n= 10292 | |  |
| --- | --- | --- | --- | --- | --- | --- | --- | --- | --- | --- | --- | --- | --- | --- | --- |
|  | n | % of diagnosed depressed | n | % of no  depression  recorded | OR (95% CI) | n | % of exposed | n | % of un-medicated | OR (95% CI) | N | % of exposed | n | % of un-medicated | OR (95% CI) |
| Population | 13,189 | 100 | 90,015 | 100 |  | 3598 | 100 | 9591 | 100 |  | 2897 | 100 | 10,292 | 100 |  |
| Anomaly or stillbirth | 486 | 3.68 | 3158 | 3.51 | 1.05 (0.96-1.16) | 134 | 3.72 | 352 | 3.67 | 1.02 (0.83-1.24) | 108 | 3.75 | 378 | 3.67 | 1.00 (0.82-1.26) |
| aL1 All anomalies | 422 | 3.20 | 2844 | 3.16 | 1.01 (0.91-1.12) | 116 | 3.22 | 306 | 3.19 | 1.02 (0.82-1.26) | 93 | 3.21 | 329 | 3.20 | 1.00 (0.79-1.27) |
| aL3 neural tube | 10 | 0.08 | 97 | 0.11 | 0.70 (0.37-1.35) | <5 |  | 6-9 |  | >1 | <5 |  | 6-9 |  | >1 |
| aL17CHD | 141 | 1.07 | 837 | 0.93 | 1.15 (0.96-1.38) | 29 | 0.81 | 112 | 1.17 | 0.69 (0.46-1.04) | 23 | 0.79 | 118 | 1.15 | 0.69 (0.44-1.08) |
| aL97Severe CHD | 24 | 0.18 | 172 | 0.19 | 0.95 (0.62-1.46) | 9 | 0.25 | 15 | 0.16 | 1.60 (0.70-3.66) | 7 | 0.24 | 17 | 0.17 | 1.46 (0.61-3.53) |
| aL 49Abdo wall defects | 16 | 0.12 | 60 | 0.07 | 1.82 (1.05-3.16) | <5 |  | 12-15 |  | >1 | <5 |  | 12-15 |  | >1 |
| al50 gastroschisis | 11 | 0.08 | 37 | 0.04 | 2.03 (1.04-3.98) | <5 |  | 7-10 |  | >1 | <5 |  | 7-10 |  | >1 |
| aL54 Renal dysplasia | 9 | 0.07 | 68 | 0.08 | 0.90 (0.45-1.81) | <5 |  | 5-8 |  | >1 | <5 |  | 5-8 |  | >1 |
| aL59 Hypospadias | 34 | 0.26 | 246 | 0.27 | 0.94 (0.66-1.35) | 12 | 0.33 | 22 | 0.23 | 1.46 (0.72-2.94) | <5 |  | 30-33 |  | >1 |
| aL 61 Limb reduction | 7 | 0.05 | 64 | 0.07 | 0.75 (0.34-1.64) | 0 |  | 7 | 0.07 | NA | 0 |  | 7 | 0.10 | NA |
| aL66 talipes equinovarus | 23 | 0.17 | 158 | 0.17 | 0.99 (0.64-1.51) | 5 | 0.14 | 18 | 0.19 | 0.74 (0.27-1.99) | <5 |  | 19-22 |  | <1 |
| al 101: Oro-facial clefts | 17 | 0.12 | 136 | 0.15 | 0.85 (0.52-1.41) | 7 | 0.19 | 10 | 0.10 | 1.89 (0.71-4.91) | 6 | 0.21 | 11 | 0.11 | 1.94 (0.72-5.25) |

Notes: Exclusions and exposures as Table 1 plus ‘not on database for 1 year either side of pregnancy’. NO6A – any antidepressant, N06AB – any SSRI. Increased time on database was associated with a diagnosis of depression and increased deprivation, but not congenital anomalies, and correlation with maternal age was low (r= -0.06). NA – unable to calculate. This is a full version of Table 7, including results for non-SSRI antidepressants (N06).
