# Supplementary material for: Selective Serotonin Reuptake Inhibitor (SSRI) Antidepressants in Pregnancy and Congenital Anomalies: Analysis of Linked Databases in Wales, Norway and Funen, Denmark
Source: PLoS One. 2016 Dec 1;11(12):e0165122. doi: 10.1371/journal.pone.0165122 (PMC5131901; doi:10.1371/journal.pone.0165122)
Supplement: S2 Appendix — (DOCX) [file pone.0165122.s002.docx]

## Appendix S2. STROBE Statement—checklist of items that should be included in reports of observational studies

|  | | Item No | Recommendation |  | Section |  |
| --- | --- | --- | --- | --- | --- | --- |
| **Title and abstract** | | 1 | (*a*) Indicate the study’s design with a commonly used term in the title or the abstract |  |  |  |
|  |  |  | (*b*) Provide in the abstract an informative and balanced summary of what was done and what was found |  | Abstract |  |
| Introduction | | | |  |  |  |
| Background/rationale | | 2 | Explain the scientific background and rationale for the investigation being reported |  | Introduction |  |
| Objectives | | 3 | State specific objectives, including any prespecified hypotheses |  | Introduction, last sentence.  Prior hypotheses are detailed under outcomes, 2^nd^ paragraph. |  |
| Methods | | | |  |  |  |
| Study design | | 4 | Present key elements of study design early in the paper |  | Methods, 1^st^ sentence. |  |
| Setting | | 5 | Describe the setting, locations, and relevant dates, including periods of recruitment, exposure, follow-up, and data collection |  | Settings, Study population, Exposure |  |
| Participants | | 6 | (*a*) *Cohort study*—Give the eligibility criteria, and the sources and methods of selection of participants. Describe methods of follow-up  *Case-control study*—Give the eligibility criteria, and the sources and methods of case ascertainment and control selection. Give the rationale for the choice of cases and controls  *Cross-sectional study*—Give the eligibility criteria, and the sources and methods of selection of participants |  | Study population  Na |  |
|  |  |  | (*b*) *Cohort study*—For matched studies, give matching criteria and number of exposed and unexposed  *Case-control study*—For matched studies, give matching criteria and the number of controls per case |  | Na |  |
| Variables | | 7 | Clearly define all outcomes, exposures, predictors, potential confounders, and effect modifiers. Give diagnostic criteria, if applicable |  | Outcomes, Exposures, Confounding (by co-exposure and by indication) |  |
| Data sources/ measurement | | 8* | For each variable of interest, give sources of data and details of methods of assessment (measurement). Describe comparability of assessment methods if there is more than one group |  | Settings |  |
| Bias | | 9 | Describe any efforts to address potential sources of bias |  | Representivity was checked, Study Population, last sentence.  Confounding (by co-exposure and by indication)  Further analyses in Wales sub-cohort. | |
| Study size | | 10 | Explain how the study size was arrived at |  | Figure 1 |  |
| Quantitative variables | | 11 | Explain how quantitative variables were handled in the analyses. If applicable, describe which groupings were chosen and why |  | Exposures, Outcomes |  |
| Statistical methods | | 12 | (*a*) Describe all statistical methods, including those used to control for confounding |  | Statistical analyses |  |
|  |  |  | (*b*) Describe any methods used to examine subgroups and interactions |  | Wales Sub-cohort |  |
|  |  |  | (*c*) Explain how missing data were addressed |  | Settings. See also the full report of the study. | |
|  |  |  | (*d*) *Cohort study*—If applicable, explain how loss to follow-up was addressed  *Case-control study*—If applicable, explain how matching of cases and controls was addressed  *Cross-sectional study*—If applicable, describe analytical methods taking account of sampling strategy | | | NA |
|  |  |  | (*e*) Describe any sensitivity analyses |  |  | NA |
| Results | | | | | |  |
| Participants | 13* | (a) Report numbers of individuals at each stage of study—eg numbers potentially eligible, examined for eligibility, confirmed eligible, included in the study, completing follow-up, and analysed | | | | Fig 1 |
|  |  | (b) Give reasons for non-participation at each stage | | | | Fig 1 |
|  |  | (c) Consider use of a flow diagram | | | | Fig 1 |
| Descriptive data | 14* | (a) Give characteristics of study participants (eg demographic, clinical, social) and information on exposures and potential confounders | | | | Tables Aa-c |
|  |  | (b) Indicate number of participants with missing data for each variable of interest | | | | Tables Aa-c |
|  |  | (c) *Cohort study*—Summarise follow-up time (eg, average and total amount) | | | | Fig 1 |
| Outcome data | 15* | *Cohort study*—Report numbers of outcome events or summary measures over time | | | | Results paragraph 1, Tables 1,3 |
|  |  | *Case-control study—*Report numbers in each exposure category, or summary measures of exposure | | | | *Table 2* |
|  |  | *Cross-sectional study—*Report numbers of outcome events or summary measures | | | | *NA* |
| Main results | 16 | (*a*) Give unadjusted estimates and, if applicable, confounder-adjusted estimates and their precision (eg, 95% confidence interval). Make clear which confounders were adjusted for and why they were included | | | | Tables 3-8 |
|  |  | (*b*) Report category boundaries when continuous variables were categorized | | | | Na |
|  |  | (*c*) If relevant, consider translating estimates of relative risk into absolute risk for a meaningful time period | | | | Results p.13 |
| Other analyses | 17 | Report other analyses done—eg analyses of subgroups and interactions, and sensitivity analyses | | | | Tables 8, D,E, Bb  Supplementary tables to the full report. |
| Discussion | | | | | |  |
| Key results | 18 | Summarise key results with reference to study objectives | | | | Paragraphs 1-3 |
| Limitations | 19 | Discuss limitations of the study, taking into account sources of potential bias or imprecision. Discuss both direction and magnitude of any potential bias | | | | Strengths and limitations |
| Interpretation | 20 | Give a cautious overall interpretation of results considering objectives, limitations, multiplicity of analyses, results from similar studies, and other relevant evidence | | | | Interpretations and Care Pathways |
| Generalisability | 21 | Discuss the generalisability (external validity) of the study results | | | | Conclusions and Implications |
| Other information | | | | | |  |
| Funding | 22 | Give the source of funding and the role of the funders for the present study and, if applicable, for the original study on which the present article is based | | | | EU |

*Give information separately for cases and controls in case-control studies and, if applicable, for exposed and unexposed groups in cohort and cross-sectional studies.

After:

von Elm E, Altman DG, Egger M, Pocock SJ, Gotzsche PC, Vandenbroucke JP. The Strengthening of the Reporting of Observational Studies in Epidemiology (STROBE) Statement: guidelines for reporting observational studies*. Ann Intern Med*. 2007;147(8):573-577.
